# Chemical fuel-driven living and transient supramolecular polymerization

**DOI:** 10.1038/s41467-019-08308-9

**Published:** 2019-01-25

**Authors:** Ankit Jain, Shikha Dhiman, Ashish Dhayani, Praveen K. Vemula, Subi J. George

**Affiliations:** 1 Supramolecular Chemistry Laboratory, New Chemistry Unit, School of Advanced Materials (SAMat), Jawaharlal Nehru Centre for Advanced Scientific Research (JNCASR), Jakkur, Bangalore, 560064 India; 2 Institute for Stem Cell Biology and Regenerative Medicine (InStem), UAS-GKVK Post, Bellary Road, Bangalore, 560065 India; 3 The School of Chemical and Biotechnology, SASTRA University, Thanjavur, 613401 India

## Abstract

Temporal control over self-assembly process is a desirable trait in the quest towards adaptable and controllable materials. The ability to devise synthetic ways to control the growth, as well as decay of materials has long been a property which only the biological systems could perform seamlessly. A common synthetic strategy which works on the biological principles such as chemical fuel-driven control over temporal self-assembly profile has not been completely realized synthetically. Here we show, we filled this dearth by showing that a chemical fuel driven self-assembling system can not only be grown in a controlled manner, but it can also result in precise control over the assembly and disassembly kinetics. Herein, we elaborate strategies which clearly show that once a chemical fuel driven self-assembly is established it can be made receptive to multiple molecular cues such that the inherent growth and decay characteristics are programmed into the ensemble.

## Abstract (web summary)

Temporal control over self-assembly processes is a desirable trait for discovering adaptable and controllable materials. Here the authors show that a chemical fuel driven system can not only self-assemble in a controlled manner, but can also result in precise control over the assembly and disassembly kinetics.

## Introduction

The field of supramolecular polymerization is undergoing a paradigm shift from simple and passive self-assembly to complex bio-inspired supramolecular polymers with explicit structural and temporal control^1–6^. Living supramolecular polymerization has emerged as a phenomenon to synthesize supramolecular polymers with controlled length and dispersity^7–14^. Contrarily, temporal programming over dynamic^15^ supramolecular materials is achieved by extending to the non-equilibrium regime^16–26^. Although these two controls are desirable, strategies to achieve them have been mostly chemically distinct. The synergy between structural and temporal control^27^ is important for the advent of supramolecular polymers to be employed as functional adaptive materials^28^. To gain this symbiosis, it is imperative that a common strategy is sought which is well-utilized by the biological realm to overcome this conundrum.

Chemical fuel-driven processes are ubiquitous in biological systems^29^. On a more microscopic view, cells use chemical fuels like adenosine triphosphate (ATP) to control their metabolic machinery in order to program the temporal tendencies of cytoskeleton proteins^30^. Control over the rate at which these proteins polymerize (grow) and depolymerize (decay) determines the temporal status of functional outcomes such as cellular motility. Actin protein is a unique example where the biological system uses a common strategy to demonstrate living and transient supramolecular polymerization^31^. An ATP(fuel)-driven transformation of non-assembling G-actin (globular) monomers to F-actin (filamentous) monomers triggers its polymerization into linear microfilament via a nucleation–elongation process. Hydrolysis of ATP to adenosine diphosphate (ADP) reverts this transformation and depolymerization occurs. In an overview, a chemical transformation determined by ATP buffers the concentration between inactive (G-forms) and active (F-forms) conformations. Hence, nature influences the kinetics of growth and decay by simply controlling the molecular cues (such as the concentration of ATP) and therefore programs the living and transient characteristics of actin polymers. In biological systems, these cues are represented by various kinds of subsidiary protein such as profilin (controls growth) and cofilin (controls decay)^32^. A unique control of these cues results in exclusive characteristics such as dynamic instability with polymers operating out-of-equilibrium. A chemical reaction controlled aggregating system, therefore, allows us to not only have a rational control over nucleated self-assembly and lead to living supramolecular polymers but also can be a biomimetic way of creating temporally dynamic transient materials^33^.

Inspired by this biological programming, herein we report a judiciously designed monomer undergoing a fuel-driven cooperative supramolecular polymerization into one-dimensional dynamic assembly. Using clever interplay of molecular cues such as the concentration of fuel and environmental factors such as pH and biocatalysts, the system is programmed to decipher fuel-driven living and non-equilibrium supramolecular polymerization to yield nanostructures with dispersity control and transient nature.

## Results

### System design

**Fig. 1:**
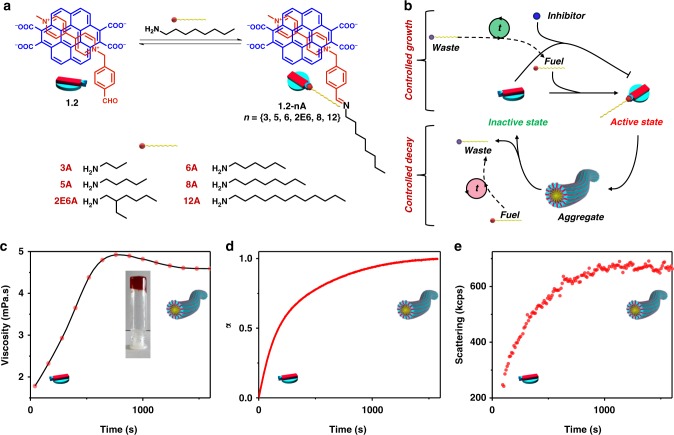
Molecular structure of the monomers and their growth kinetics. **a** Molecular structures of a charge-transfer complex (**1.2**) between aromatic donor **1**, and benzaldehyde substituted viologen acceptor **2**, along with various alkyl amines used in this study. **b** Schematic illustration of the reaction-driven temporal control of supramolecular polymerization of **1.2**. **c**–**e** Time-dependent evolution of viscosity trend, absorption trace at 515 nm, and scattering respectively monitored for the kinetics of formation of **1.2-8A**. Inset in **c** is the photograph of hydrogel of **1.2**-**8A** (10 mM). α is the extent of self-assembly (For **c**–**e**: [**1**] = [**2**] = [**8A**] = 1 mM, pH = 11.0)

In this work, we intend to develop and study a chemical reaction-driven supramolecular system that not only shows living supramolecular polymerization but can also be synthesized transiently by regulating various molecular cues. Our group has designed supramolecular amphiphiles and exploited their electronic prowess on a device structure^34,35^. One system that stands out of the many in terms of its materials applications is the charge-transfer (CT) amphiphile comprising of tetra potassium coronene salt **1** as donor and amphiphilic methyl viologen as acceptor^36^. The supramolecular polymers comprising of this CT amphiphile shows excellent conducting properties. However, this system lacks dispersity control and its mechanistic aspects could not be studied owing to a very fast self-assembly and hence functional control was lacking. For a better structural and temporal control, herein we have employed a dynamic imine bond chemistry to kinetically control the formation of this supramolecular polymer^37–39^. The design involves the conversion of non-assembling CT complex (**1.2**, inactive/dormant state) between aromatic donor **1** and benzaldehyde substituted viologen acceptor **2** (Fig. 1a, for synthesis refer Supplementary Method and Supplementary Figure 1 and 2) into a self-assembling CT amphiphile (**1.2-nA**, active state) via an in situ kinetically controlled imine bond formation with an alkyl amine (nA, fuel) similar to inactive and active states of protein monomers in biological assemblies which can be triggered by a fuel. Various amines have been investigated in this study to leverage the supramolecular polymerization tendencies and explore the mechanism of temporal supramolecular polymerization.

### Inter-dependency between reactivity and growth

Since the covalent bond formation is a kinetic process, it is envisaged that the resulting temporal control would be a two-way street. By this dual control, we refer to the fact that the extent of self-assembly would depend on the amount of amine present. On the other hand, the equilibrium constant of the imine formation would be driven by the self-assembling species (which refers to the fact that alkyl chains incapable of forming aggregates would consume less **1** and vice versa). Studies were performed in an aqueous buffer of pH 11. This was fixed due to a combination of factors as pH 11 gave maximum imine formation and minimal side reactions in the time period of investigation. To probe this interdependence of reactivity and growth, we performed sequential ^1^H-NMR and UV–vis studies (Supplementary Figure 3). The extent of aldehyde consumption in case of ^1^H-NMR and the supramolecular polymerization induced changes in the CT band, were hence tabulated with various alkyl amines (Supplementary Figure 3b,d). Peaks in ^1^H-NMR remained fairly similar from blank up until *n*-hexylamine (**3A**, **5A**, and **6A**). Beyond this the addition of 2-ethyl hexylamine (**2E6A**), *n*-octyl amine (**8A**), and *n*-dodecyl amine (**12A**) showed remarkable changes in the pattern of spectra; signifying a self-assembly process (Supplementary Figure 3c). Concurrently, higher homologues of alkyl amine (**2E6A**, **8A**, **12A**) showed much higher aldehyde consumption than smaller homologues of alkyl amines (**3A**, **5A**, **6A**). This result propagates the idea of an assembly-driven equilibrium. As the intensity of the CT band is inversely proportional to the proximity of donor and acceptor molecules, i.e., smaller the distance, higher is the CT strength, hence upon self-assembly the distance between the donor and acceptor decreases and a significant enhancement in absorbance of the CT band should be observed. Considering this scenario, the absorption spectra of each of the samples were recorded and their absorbance at 515 nm (to signify the increase in absorbance) was tabulated against the alkyl amine used (Supplementary Figure 3b). The components of the mixture remained in solution throughout the experiment and hence negated any scattering-induced effects. The results obtained were in coherence with the ^1^H-NMR data. Longer alkyl chains (**2E6A**, **8A**, **12A**) showed a much higher tendency to assemble than smaller alkyl amines (**3A**, **5A**, **6A**). Hence, longer alkyl chains have enough amphiphilicity to undergo supramolecular polymerization and thus they pull the imine bond formation equilibrium and a high extent of imine formation and self-assembly is observed. In contrast, smaller alkyl chains form imine only by their reactive capacity and are not driven to higher consumption due to low potency of aggregation.

### Elucidation of growth characteristics

Moving ahead we wanted to analyze the temporal control that this system exhibits and for doing the same we selected **8A** as the representative amine showing a high degree of imine formation and self-assembly (**12A** showing a different mechanism has been discussed in parallel in the Supporting Information). The kinetics of growth was initially followed by time-dependent changes in solution viscosity (Fig. 1c). A clear growth profile observed validates the in situ self-assembly process. This could be further elucidated by the fact that at higher concentration (10 mM) a gel was formed suggesting the existence of linear assembly of **1.2-8A** (inset, Fig. 1c). The kinetics of **1.2-8A** formation was also followed by ^1^H-NMR (aldehyde consumption), UV–vis absorption (CT band at 515 nm), and dynamic light scattering (kcps measurement) (Supplementary Figure 4, Fig. 1d, e). All the three experimental techniques displayed a time-dependent evolution with similar rate constants (^1^H-NMR: 2 × 10^−3^ s^−1^, absorbance: 3 × 10^−3^ s^−1^, and dynamic light scattering (DLS): 3.2 × 10^−3^ s^−1^) suggesting that imine formation is intertwined with the growth of supramolecular polymer of **1.2-8A**. These rate constants are a cumulative measure of a nucleation and elongation process which has been detailed with further studies in this work^40^. The observations are important as this is a prerequisite for a chemical fuel-driven assembly. Concentrations of in-active and active states should be controlled by the chemical fuel. This would further prove vital in controlling the characteristics of growth and decay in these assemblies (vide infra).

**Fig. 2:**
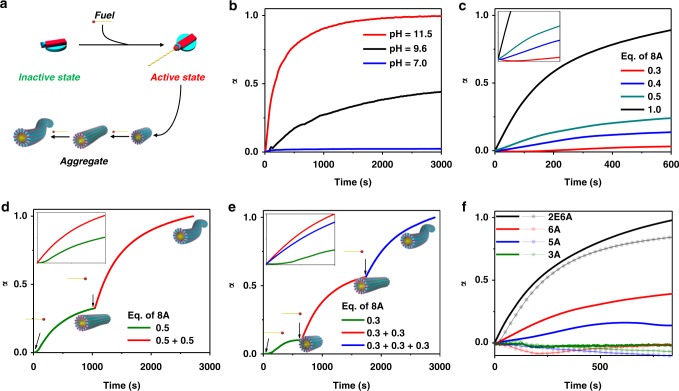
Characteristics of alkyl amine-driven supramolecular polymerization. **a** Schematic representation of the extent of self-assembly dependent on the concentration of **8A**. Absorption trends at 515 nm of **1.2-8A** growth **b** at various pH, **c** with various equivalents of **8A**, **d** with 0.5 eq. of **8A** added in subsequent batches, **e** with 0.33 eq. of **8A** added in subsequent batches, **f** with 0.5 eq. of various amines after seeding with 0.5 eq. of **8A**. The translucent overlays are profiles of unseed growth or respective nAs. (Insets for **c**, **d**: respective graphs on linear scale to easily elucidate the change in lag phase and kinetics.) Arrows indicate the addition of respective **nA**. (For **b**, **c**, **f**: [**1**] = [**2**] = 1 mM, 50 mM pH = 11.0 buffer; for **d**, **e**: [**1**] = [**2**] = 0.5 mM, 50 mM pH = 11.0 buffer)

To further elucidate the growth characteristics, a key experiment would be to study the pH dependence of growth. This is because the imine bond formation is highly dependent on the nucleophilicity of the amine and hence is a factor of pH. Time-dependent absorption trends at 515 nm with varying pH from 7 to 9.6 to 11 suggest that higher the pH, higher and faster is the conversion due to the presence of higher amount of deprotonated amine (Fig. 2b). We further added variable equivalents of **8A** into a solution of **1.2**, and as expected, with higher equivalents of **8A** higher and faster formation of **1.2-8A** was observed (Fig. 2c). The trend of longer supramolecular polymers formation with higher amounts of **8A** was observed in DLS measurements, corroborating with the UV–vis trends (Supplementary Figure 5). Interestingly, the absorption trends observed were visibly sigmoidal in nature pointing out towards a nucleation–elongation growth mechanism (inset, Fig. 2c). For further insights, the kinetic growths were fit to Finke’s two-state model and nucleation constants were extracted^41^. The nucleation rate constant (*k*_*n*_) was observed to increase with the increased equivalent of **8A** added (from 9.6 × 10^−4^ s^−1^ for 0.3 eq. to 4.4 × 10^−3^ s^−1^ for 1.0 eq.).

We next envisaged to utilize the chemical reaction-driven cooperative supramolecular polymerization to study the seeding ability of these kinetically grown supramolecular polymers. Contrary to convention, in the present case, the process of addition of seed to fresh monomer solution is different as we can form the seed in situ by addition of **8A** in batches instead of adding 1 eq. as has been done in previous experiments (Supplementary Figures 6–8). To a solution of inactive CT complex **1.2**, **8A** was added in two subsequent batches of 0.5 eq. each (Fig. 2d). First addition evolved in a sigmoidal trend with *k*_*n*_ being 5.8 × 10^−4^ s^−1^. The second batch however was devoid of the sigmoidal characteristics. Similar observations were made as **8A** was added in three subsequent batches of 0.33 eq. each. The first growth had sigmoidal trends (*k*_*n*_ = 1.1 × 10^−4^ s^−1^) which transformed into much faster growth in subsequent additions (Fig. 2e, Supplementary Figure 10). It should be noted that an increase in the degree of aggregation is smaller in first batch addition as compared with subsequent additions. We believe that the difference in alpha during subsequent additions is a manifestation of imine formation being uniquely linked to nucleation and aggregation characteristics. Since the subsequent additions happen in the presence of nuclei their conversions are higher. A similar phenomenon is observed while variable amine addition in batches (vide infra). To further strengthen the argument, we performed the batch growth study also through scattering (Supplementary Figure 9). We observed a clear size increase on subsequent addition of **8A**. This change in size as well as non-sigmoidal growth of the incoming monomer signifies that as the seed is formed subsequent monomers are recruited at the interface to stabilize the interfacial energy. We also took advantage of the variability in alkyl chains and excavated deeper into understanding the seeded aggregation process (Fig. 2f). To a mixture of **1.2-8A** seed, various amines were added (Fig. 2f, Supplementary Figure 11), their growth profiles were subsequently compared to one with un-seeded amines. Remarkably, we observed that nucleation rates were visibly enhanced in most cases. These experiments cumulatively prove convincingly that **1.2-nA** shows a seeded behavior. Seeded experiments with higher alkyl chains (**12A**) were also performed and showed similar characteristics (Supplementary Figures 12–20).

### Morphological analysis of supramolecular polymers

**Fig. 3:**
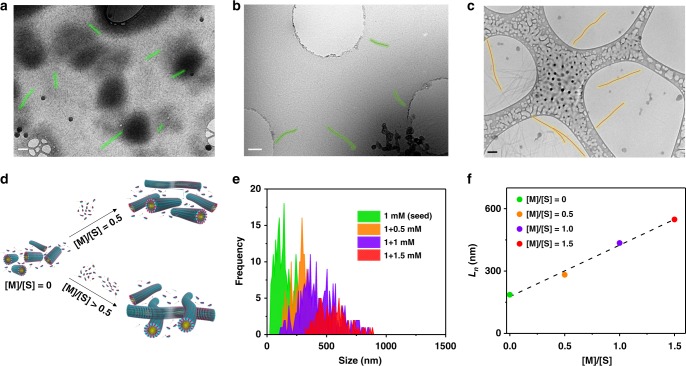
Investigation of controlled growth by cryo-TEM. **a**–**c** Cryo-TEM images of **1.2-8A** with varying amounts of monomer addition onto a seed. [**a** seed (1 mM of **8A**, [**1.2**] = 1 mM), **b** seed (1 mM of **8A**) + monomer (0.5 mM of **8A**) ([**1.2**] = 1.5 mM), **c** seed (1 mM of **8A**) + monomer (1.0 mM of **8A**) ([**1.2**] = 2 mM)]. **d** Schematic representation of seeded growth. **e** Frequency distribution of number average lengths (*L*_*n*_) obtained from the cryo-TEM images. **f** Linear trend between *L*_*n*_ and monomer to seed ratio. Scale: **a** 100 nm, **b** 200 nm, **c** 100 nm. Green and brown overlays in **a**–**c** point out some of many such fibers

Having established the growth mechanism spectroscopically, we went ahead to seek further proof if this seeded supramolecular polymerization has the structural characteristics of a living supramolecular polymer. Field emission scanning electron microscopy (FE-SEM) and transmission electron microscopy (TEM) of the dried samples of **1.2-8A** showed the presence of one-dimensional structures (Supplementary Figure 21a,b). These fibers, however, were the bundles of molecular assemblies as suggested by their width which ranges from 30 to 100 nm. On the other hand, cryo-TEM analysis clearly showed molecular fibers of almost constant width of 6.6 nm (Supplementary Figure 21c-e) which corroborated with the cylindrical micellar packing that has been observed in covalent homologues^35^. Cryo-TEM experiment also clearly proved that the assembly is indeed linear and thus justifying our molecular design parameters. Furthermore, to investigate the structural control in these supramolecular polymers, we carried out cryo-TEM studies on nucleated **1.2-8A** samples where the ratio of incoming monomers [M] to the seed [S] varied from 0 to 1.5. A frequency statistics was done on the obtained lengths and their number (*L*_*n*_) and weighted average (*L*_*w*_) was calculated and polydispersity index (PDI) was calculated as *L*_*w*_/*L*_*n*_ (Fig. 3, Supplementary Figure 22). Calculated *L*_*n*_ was in linear correlation with the ratio between monomer and seed concentrations ([M]/[S]) and the PDI calculated was as low as 1.1. These statistics suggest that supramolecular polymers are fairly monodisperse, hence suggesting that the seeded growth of **1.2-8A** is indeed living in nature.

### Controlling the growth kinetics of supramolecular polymerization through ligand dilution

**Fig. 4:**
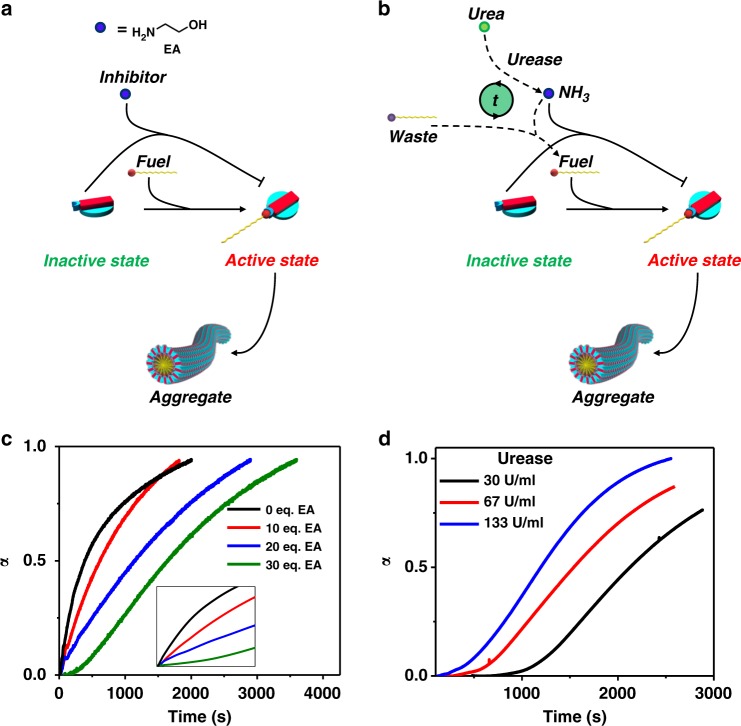
Temporal control over nucleation. **a** Schematic representation of delay in growth through ligand dilution and delayed activation. Ligand dilution represents ethanol amine (**EA**) acting as a competitive inhibitor. **b** Schematic representation of delay in growth through delayed pH change using urease. **c** Absorbance trend, at 515 nm, of **1.2-8A** growth with various equivalents of **EA** (inset: same graph on linear scale to easily elucidate the change in lag phase). ([**1**] = [**2**] = [**8A**] = 1 mM, 50 mM pH = 11 buffer). **d** Absorbance trend, at 515 nm, of **1.2-8A** growth with variation of urease concentration ([**Urea**] = 400 mM, [**1**] = [**2**] = 1 mM, [**8A**] = 5 mM, 200 mM pH = 7 buffer)

Although imine bond formation assisted in living supramolecular polymerization, we envisaged to further control the supramolecular polymerization process by delaying the nucleation process with an action similar to profilin on actin filaments^32^. This competition of substrates has largely been limited to libraries which do not form extended assemblies^42–44^. Thus, considering the uniqueness of current system it was hypothesized that a competition between two amine substrates, one with higher reactivity but no aggregation (ethanol amine (**EA**)) and the other with higher aggregation tendencies (*n*-octyl amine (**8A**)) could result in a retarded growth of supramolecular polymers (Fig. 4a, ligand dilution). As a preliminary experiment to a completely grown **1.2-8A** solution, 60 eq. of **EA** was added and an immediate disassembly was observed, establishing the fact that addition of **EA** can result in the formation of non-assembling imine species (Supplementary Figure 23). Going ahead with the controlled growth rate experiment, various equivalents of **EA** (0–30 eq.) were thus added in tandem with **8A** into a solution containing **1.2** (Fig. 4c).

Twenty-five times retardation in the growth was observed (for 30 eq., *k*_*n*_ = 1.8 × 10^−4^ s^−1^) compared with the blank aggregate growth that did not contain **EA** (*k*_*n*_ = 4.4 × 10^−3^ s^−1^) (Supplementary Figure 24). We believe this slowing down is because of the fact that an additional equilibrium has been introduced in the solution with the addition of **EA** which is competing for **1.2** population. **EA** being in excess and exhibiting higher reactivity consumes **1.2** population to form **1.2-EA**, however as **1.2-8A** begins to form and starts to polymerize there is an attrition in **1.2-EA** population which gives way to a lesser reactive product with **8A** but more energetically favorable due to self-assembly which **1.2-EA** cannot undergo. Temporal control that can be exerted on the growth of extended supramolecular polymers exemplifies the simplicity and scope of development in the present system.

### Controlling the growth kinetics of supramolecular polymerization through delayed activation

Furthermore, since here we use alkyl amines (**nA**) with pKa around 8.3, change of pH in this window can lead to varying percentages of protonated and pristine amines, in turn becoming a tool for reactivity and self-assembly control. Therefore in the present system, a temporal and gradual increase in pH would retard the fast nucleation by slow activation of monomers. Urease is an enzyme that acts on urea at low pH to yield ammonium hydroxide that increases the pH temporally. Hence, coupling fuel-driven growth to urease would add an extra kinetics of pH change and this finite time of activation of amines would thus decide the retardation produced in the nucleation of **1.2-8A** supramolecular polymers (Fig. 4b, delayed activation)^45^. Additionally, ammonia can also have some imine formation and thus delay the growth as in the case of EA giving two-step inhibition of spontaneous growth (Supplementary Figure 26b). Various pH and control UV–vis spectra and trends were recorded to elucidate the appropriate concentrations for key parameters such as **8A**, urea, urease and the buffer solution (Supplementary Figures 25–27). For supramolecular polymerization experiments, urea and buffer concentration was thus fixed to be 400 and 200 mM, respectively. Enzyme concentration was varied from 30 to 133 U/ml. Initial pH of solution was also changed from 4 to 7 as **1** is susceptible to protonation and is not stable in solution below that pH. Due to this fact, a significant amount of delay time is lost, but this was necessary to ensure a comparatively homogenous starting solution. We then proceeded to see the changes in UV absorption spectra at 515 nm that urease system brings about in **1.2-8A** growth (Fig. 4d). With variation of enzyme concentration, the growth could be delayed up to 1000 s (*k*_*n*_ values of 1.4 × 10^−4^ s^−1^, 8.2 × 10^−5^ s^−1^, and 2.4 × 10^−5^ s^−1^ for enzyme concentrations of 133, 67, and 30 U/ml respectively) (Supplementary Figure 28). This delay of course was also dependent on **8A** concentration again reiterating the fact that this assembly is due to imine formation alone (Supplementary Figure 29a). Since the time course of this growth results in scattering, the kinetics were monitored at a red-shifted wavelength (650 nm) (Supplementary Figure 29b). The growth kinetics obtained here also nearly matched the kinetics of CT band growth suggesting a clear dependence. We also analyzed the morphology of the resulting final solution with FE-SEM (Supplementary Figure 30). We saw arrays of long, bundled, fibers spanning a few micrometers reiterating the linear growth model as described in the previous section. The reason behind bundling is perhaps the weak secondary interaction of supramolecular polymers with urea and released ammonia.

We thus were successful in showing, through ligand dilution and delayed activation as strategies that external molecular cues can seamlessly control the growth of a chemical reaction drive aggregation reminiscent of profilin like control over actin assembly^32^.

### Elucidating transient supramolecular polymerization

As we aim towards attaining living and transient characteristics from a single system, we next attempted to control the decay of these supramolecular polymers. Since the assembly is governed by imine formation, we again took advantage of the fact that these bonds are highly pH sensitive. Thus, to disassemble the supramolecular polymer, the pH needs to be decreased from basic to neutral (Supplementary Figure 31) and hence a stimulus that can not only change pH gradually but also is kinetically delayed, is required. This delay is necessary as fast immediate pH quencher would not allow any growth to occur as the time required to reach the neutral pH would be too less. To fulfill this scenario, we employed saponification of esters as a potential chemical agent^45–48^. Esters in the presence of base consume hydroxyl ions to facilitate their hydrolysis into carboxylate (until the pKa is reached) and alcohol and thereby decreasing the pH. Various esters hydrolyze at different rates depending on their structural and electronic constraints, thus the abundance of structural diversity of esters allow us to choose from a plethora of molecules such that the selected ester fits the kinetic requirement. For the present study, we choose gamma-Butyrolactone (**γ-BL**), epsilon-Caprolactone (**ε-CL**), and beta-Butyrolactone (**β-BL**) as hydrolyzing esters as their saponification reaction, which results in decrease in pH, is fairly gradual making it ideal for use (Supplementary Figure 31).

To see the changes in the UV–vis absorption spectra, we followed the kinetics of **1.2-8A** and recorded individual spectra with time lapse (Supplementary Figure 32). As a prototype, the experiment was done with 500 mM of **γ-BL** with starting pH = 11. It can be clearly seen that the absorbance increases with the maxima shifting from 492 to 505 nm signifying the growth phase. Consequently, it starts to decrease gradually and finally comes back to similar absorbance with maxima at 500 nm, signifying the disassembly/decay phase. This process was then followed at 515 nm for all the three lactones (Supplementary Figure 33). This clearly suggests a growth phase and a decay phase. The lifetime of the decay phase could not only be modulated with the concentration of lactones but also with the kind of lactone. For **γ-BL** on one hand the lifetime can be modulated from less than 100 s to roughly around 500 s. However, as the lactone is varied this lifetime can be increased to roughly 950 s (**β-BL**, **ε-CL**). Having established that the transient growth can be achieved we followed the **1.2-8A** aggregation with viscosity and DLS containing **γ-BL** as a model lactone (Supplementary Figure 34). Both scattering and viscosity measurements reiterated the trend of a transient behavior with the lifetime of the transient supramolecular polymer varying clearly with changing concentrations of the lactone. We further envisaged that since the decay is due to protonation of the **8A** and introduction of few microliters of conc. NaOH solution might refuel the ensemble towards another cycle of transiency (until the lactone is exhausted) (Supplementary Figure 35). We performed this experiment with 500 mM of **γ-BL** and 500 mM of **ε-CL** to signify the various lifetimes and cycles of transiency that can be achieved. In the case of 500 mM of **γ-BL**, 31.25 µl of 1.6 M NaOH was introduced at an intermediate point of on-going decay. The process was monitored by absorption (at 515 nm) as well as DLS. Both absorption and DLS trends clearly show that indeed these assemblies can be refurbished, and once revived, again undergo controlled decay. The first cycle was clearly faster than the second one. We hypothesize this is due to the concentration of lactone has decreased from the initial point as we saw previously a decreased concentration results in a slower transient cycle. Cryo-TEM studies were done at different time points of an evolving solution of **1.2-8A** with 500 mM **ε-CL** to get a consolidated proof of this disassembly (Supplementary Figure 36–38). The images clearly show the polymerization and eventually depolymerization of fibers further validating our hypothesis.

**Fig. 5:**
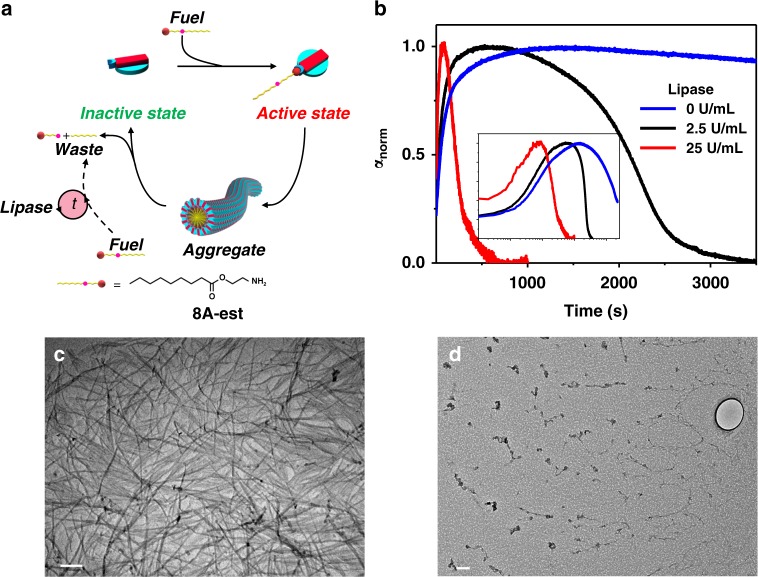
Inherent transience in supramolecular polymerization. **a** Schematic representing the prospective inherent transient self-assembly affected by lipase. **b** Temporal profile of **1.2-8A-est** growth at various concentrations of lipase ([**1**] = [**2**] = 1 mM, [**8A-est**] = 2 mM, pH = 11). (Inset shows changes with time in log scale.) **c**, **d** Cryo-TEM images of **1.2-8A-est** at 2, 24 h respectively. (pH = 11, [**1.2**] = 1 mM, [**8A-est**] = 2 mM, [**Lipase**] = 0 U/ml). Scale for **c**, **d**: 200 nm

Having established this, the transiency in **1.2-8A** through an external deactivator (lactones), we sought to add further to the system and devise molecules for inherent or internal transience in supramolecular polymers. This design would be significantly closer to the biological systems like actin which depolymerize due to inherent ATP hydrolyzing activity. For this purpose instead of regular **nA**s, we designed a special amine (**8A-est**) with the labile ester group embedded into the alkyl chain (Fig. 5a). We believe that this induction of the labile ester group would be hydrolysable in basic imine-forming conditions, resulting in shortening of the alkyl chain and thus eventually disassembling thus obtaining an out-of-equilibrium supramolecular polymerization. Furthermore, this dynamic instability can be affected by external molecular cues such as ester hydrolyzing enzymes such as lipases.

To a solution of **1.2**, **8A-est** was introduced and inherently at pH = 11 transiency, albeit slow, was clearly visible. The inherent lifetime of the transient state could further be modulated from few hours, to few minutes, and finally few seconds by addition of varying amounts of lipase (Fig. 5b, Supplementary Figure 39, 41). This transient transition could also be seen clearly in a cryo-TEM analysis (Fig. 5c, d, Supplementary Figure 40).

## Discussion

In this study, we have developed a supramolecular polymer whose self-assembly is driven by a chemical reaction. We have further categorically shown the interdependency of reactivity and growth of the supramolecular polymer. Spectroscopic studies have shown that this system grows via a fuel-driven cooperative and seeded mechanism. We further show that these reaction-driven assemblies can undergo living supramolecular polymerization with critical control over its length and dispersity. We have also shown that the reaction and subsequently the supramolecular polymerization could be retarded by molecular cues that compete with the imine-forming reaction. The system has been further developed to show transient supramolecular polymerization and out-of-equilibrium growth. Here, we have shown that a common biomimetic fuel-driven strategy can be used to reconcile structural and temporal control over supramolecular polymers. We believe this strategy, which as shown in this study can be easily coupled to external molecular cues, can be crucial in developing adaptive and temporally more complex materials^49,50^.

## Methods

### Sample preparation

All experiments were done at 25 °C.

### Seeded assembly

Stock solutions of **1** and **2** were made in 50 mM, pH = 11, sodium phosphate buffer. Appropriate volumes of the individual stocks were mixed and diluted with buffer to create 1 mM solution containing ground state charge transfer complex of **1.2**. Stock solutions of respective amines (**nA**) were made in DMSO and introduced, in equivalence-dependent volumes, to the aforementioned **1.2** solution. Net dilution was 0.1% for 1.0 equivalent of **nA**. Immediately after the addition, the solution was slightly shaken to ensure mixing and UV traces were thus measured. For seeding experiments, similar addition was done in parts and thus monitored.

### Ligand dilution

Stock solutions of **1** and **2** were made in 50 mM, pH = 11, sodium phosphate buffer. Appropriate volumes of the individual stocks were mixed and diluted with buffer to create 1 mM solution containing ground state charge transfer complex of **1.2**. To this, a DMSO stock of **EA** was added, mixed, and after 5 min (time given for pre-formation of imines), a stock solution of **nA** (made in DMSO) was introduced into the solution. After slight shaking, sample was immediately monitored through UV absorption. Net dilution was less than 1%.

### Delayed activation

For enzymatic delayed activation, starting pH was 7. Variable buffer (sodium phosphate), urea, urease concentrations were tried. The sequence of addition was as follows. To a solution of **1.2** (1 mM), urea was added followed by appropriate volumes of **nA**. Urease was the last component. After shaking the solution slightly for mixing, it was monitored for analysis.

### Delayed deactivation

Stock solutions of **1** and **2** were made in 50 mM, pH = 11, sodium phosphate buffer. Appropriate volumes of the individual stocks were mixed and diluted with buffer to create 1 mM solution containing ground state charge transfer complex of **1.2**. To this a DMSO stock of **lactone** was added, followed almost immediately by respective volume of nA stock in DMSO. Net dilution was less than 1%. After slight shaking, sample was immediately monitored through UV absorption.

### Enzyme-coupled transience

Stock solutions of **1** and **2** were made in 50 mM, pH = 11, sodium phosphate buffer. Appropriate volumes of the individual stocks were mixed and diluted with buffer to create 1 mM solution containing ground state charge transfer complex of **1.2**. To this a DMSO stock of **8A-est** was added, followed almost immediately by respective volume of lipase stock in 50 mM pH = 11 buffer. Net dilution was less than 1%. After slight shaking, sample was immediately monitored through UV absorption.

Detailed synthesis and characterization of compounds and measurement protocols have been provided in Supplementary Information.

## Supplementary information

Supplementary Information

## Data Availability

All data supporting the findings are available in the article as well as the Supplementary Information files from the authors on reasonable request.
