# Supplementary material for: Chemical fuel-driven living and transient supramolecular polymerization
Source: Nat Commun. 2019 Jan 25;10:450. doi: 10.1038/s41467-019-08308-9 (PMC6347607; doi:10.1038/s41467-019-08308-9)
Supplement: Supplementary file 1 — Supplementary Information [file 41467_2019_8308_MOESM1_ESM.pdf]

Chemical Fuel-Driven Living and Transient Supramolecular Polymerization

Ankit Jain,[1] Shikha Dhiman,[1] Ashish Dhayani,[2,3] Praveen K. Vemula[2]* and Subi J. George[1]*

[1] Supramolecular Chemistry Laboratory, New Chemistry Unit, School of Advanced Materials (SAMat), Jawaharlal Nehru Centre for Advanced Scientific Research (JNCASR), Jakkur, Bangalore 560064, India.

[2] Institute for Stem Cell Biology and Regenerative Medicine (inStem), UAS-GKVK post, Bellary Road, Bangalore 560065, India.

[3] The School of Chemical and Biotechnology, SASTRA University, Tamil Nadu, India

*email: george@jncasr.ac.in, praveenv@instem.res.in

Supplementary Methods

n-propyl amine, *n*-pentyl amine and *n*-octyl amine were procured from Spectrochem. *n*-hexyl amine (99%) and *n*-dodecyl amine (98%) were ordered from Sigma Aldrich. 2-Ethyl hexyl amine (99%) was procured from Acros organics. Ethanol amine, gamma-Butyrolactone (99%), epsilon-caprolactone (97%) and beta-butyrolactone (98%) were procured from Sigma Aldrich. Urea and Urease (From kidney beans, 380 U/mg) was ordered from SRL. All the chemicals were not further purified and used as such. Phosphate buffer solutions of various pHs was made according to the reported protocol.[1]

Synthesis

1 and **3** were synthesized according to the reported procedures and sufficiently characterized.[2,3]

2:

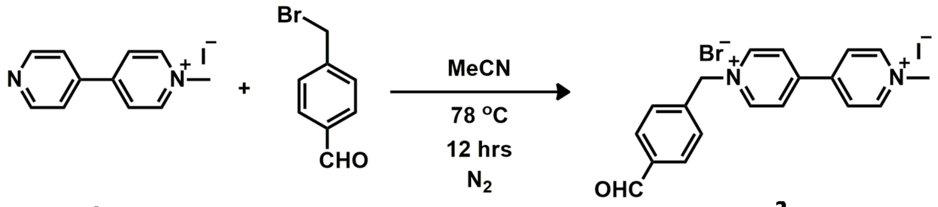

Supplementary Figure 1. *Synthetic scheme for synthesis of **2**.*

Synthesis of **2**:

A and **B** were dissolved in dry acetonitrile and allowed to react for 12 hours under nitrogen atmosphere. The resultant orange colour precipitate was filtered and subsequently washed with chloroform and acetonitrile. The precipitate was then dried and analysed (98% yield).

^{1}H NMR (400 MHz, D_2O): δ 10.03 (s, 1H), 9.25 (d, 2H, J = 6.88 Hz), 9.10 (d, 2H, J = 6.80 Hz), 8.64 (d, 2H, J = 6.84 Hz), 8.58 (d, 2H, J = 6.72 Hz), 8.09 (d, 2H, J = 8.20 Hz), 7.45 (d, 2H, J = 8.2 Hz), 6.11 (s, 2H), 4.55 (s, 3H); ^{13}C NMR (100 MHz, D_2O): δ_c 195.6, 150.8, 149.6, 146.3, 145.8, 138.9, 136.6, 131.0, 129.7, 127.3, 126.8, 64.2, 48.4; ^{13}C DEPT 135 NMR δ_c (100 MHz, D_2O): 195.6 (+), 146.3 (+), 145.8 (+),131.0 (+), 129.7 (+), 127.3 (+), 126.8 (+), 64.2 (-), 48.4 (+); HRMS (ESI): m/z: calcd for M i.e. $C_{19}H_{18}BrIN_2O$: 495.9647, for $[M\text{-}Br\text{-}I\text{-}H]^+$ i.e. $C_{19}H_{17}N_2O$: 289.1335, found $[M\text{-}Br\text{-}I\text{-}H]^+$: 289.2403.

A and **B** were synthesized and adequately analysed according to reported procedures.[4,5]

8A-est:

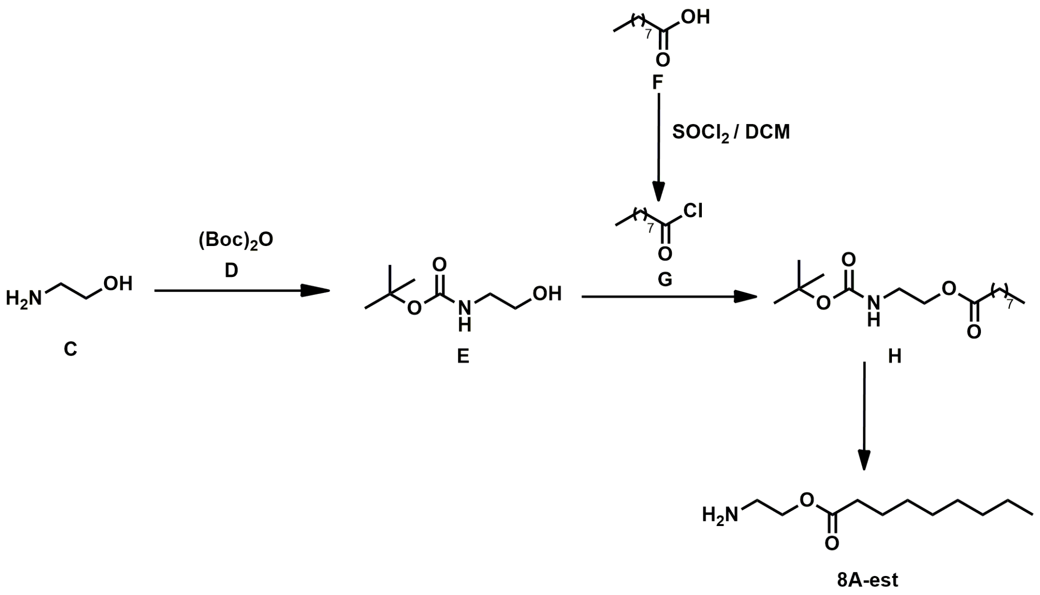

Supplementary Figure 2. *Synthetic scheme for synthesis of* ***8A-est****.*

Synthesis of tert-butyl (2-hydroxyethyl)carbamate **(E):**

Di-tert-butyl dicarbonate (8.579 g, 39.31 mmol) was added to ethanolamine (2 g, 32.76 mmol) at 0 °C, and the mixture was allowed to stir for 3h. The completion of the reaction was monitored by TLC. The reaction was diluted with water and extracted twice with ethylacetate. The crude product was purified by column chromatography using 20% ethylacetate in hexane with 60-120 mesh silica gel to get 4g of (75.9%) pure tert-butyl (2-hydroxyethyl)carbamate (**E**).

^{1}H NMR (600 MHz, $CDCl_3$): δ 4.97 (1H, bs), 3.72-3.71 (2H, m), 3.31-3.30 (2H, m), 1.50 (9H, s); ^{13}C NMR (150 MHz, $CDCl_3$): δ_c 152.87, 82.04, 62.31, 42.75, 27.30; MS: m/z: calcd for M i.e. $C_7H_{15}NO_3$: 161.1052, found $[M+1]^+$ = 162.2

Synthesis of nonanoyl chloride **(G):**

To a solution of nonanoic acid (**F**) (1.0 g, 6.32 mmol) in dichloromethane (20 ml) thionyl chloride (2.25 g, 18.97 mmol) and two drops of catalytic DMF were added at 0 °C. The reaction mixture was refluxed for 3h. The completion of the reaction was monitored by TLC. The solvents were removed under reduced pressure to get nonanoyl chloride (**G**). The crude product was directly used for next step.

Synthesis of 2-((tert-butoxycarbonyl)amino)ethyl nonanoate **(H):**

To a solution of tert-butyl (2-hydroxyethyl)carbamate (**E**) (1.0 g 6.20 mmol) and triethyl amine (1.25 g, 12.4 mmol) in dichloromethane (25 ml), nonanoyl chloride (1.1 g, 6.20 mmol) was added dropwise at 0 °C. The reaction mixture was stirred at room temperature for 4h. After completion of the reaction, it was poured into ice. The organic layer was separated and washed with saturated sodium bicarbonate, brine and dried over anhydrous sodium sulphate, evaporated under reduced pressure to

get the crude product. The crude product was purified by column chromatography using 40% ethylacetate in hexane with 60-120 mesh silica gel to get 0.8g (43%) pure 2-((*tert*-butoxycarbonyl)amino)ethyl nonanoate (**H**).

^{1}H NMR (600 MHz, $CDCl_3$): δ 4.78 (1H, bs), 4.15-4.13 (2H, m), 3.41-3.39 (2H, m), 2.34-2.32 (2H, m), 1.64-1.62 (2H, m), 1.46 (9H, s), 1.32-1.20 (10H, m), 0.91-0.88 (3H, t, J=7.2Hz); ^{13}C NMR (150 MHz, $CDCl_3$): δ_c 173.39, 155.36, 79.17, 63.00, 39.30, 33.75, 30.99, 28.36, 28.30, 27.30, 24.52, 22.02, 13.56; MS: m/z: calcd for M i.e. $C_{16}H_{31}NO_4$: 301.2253, found $[M+1]^+$ = 302.2

Synthesis of 2-aminoethyl nonanoate **(8A-est):**

To a stirred solution of 2-((*tert*-butoxycarbonyl)amino)ethyl nonanoate (**H**) (0.5g 1.66mmol) in dichloromethane (20 ml), trifluoroacetic acid (10 ml) was added at 0 ^{0}C. The reaction mixture was stirred at room temperature for 12 h. After completion of the reaction, it was poured into ice. The organic layer has been separated and washed with saturated sodium bicarbonate, brine and dried over anhydrous sodium sulphate, evaporated under reduced pressure to get the crude product. The crude product was purified by column chromatography using 10% methanol in chloroform with 60-120 mesh silica gel to get 0.2 g (60%) pure 2-aminoethyl nonanoate (**8A-est**).

^{1}H NMR (600MHz, DMSO-d_6): δ 7.70 (2H, bs), 4.17-4.16 (2H, t, J=5.4 Hz), 3.07-3.05 (2H, t, J=5.4 Hz), 2.34-2.31 (2H, m), 1.54-1.52 (2H, m), 1.31-1.20 (10H, m), 0.87-0.85 (3H, t, J=7.2Hz); ^{13}C NMR (150 MHz, $CDCl_3$): δ_c 172.84, 60.76, 38.12, 33.28, 31.24, 28.69, 28.56, 28.49, 24.22, 22.09, 13.97; MS: m/z: calcd for M i.e. $C_{11}H_{23}NO_2$: 201.1729, found $[M+1]^+$ = 202.2

Measurements

Photo-physical measurements:

Electronic absorption spectra were recorded on a Perkin Elmer Lambda 900 UV-Vis-NIR Spectrometer. Time dependent traces were recorded at 515 nm for all amines except **12A** which was recorded at the scattering wavelength of 750 nm. **nA** stock solution was made in DMSO and was introduced by a pipette in an aqueous solution of pH = 11.0 containing **1** and **2**. The dilution was maximum 0.1%. All growth profiles have been normalized between 0 and 1 to refer to degree of aggregation (α). An important point however is that extent of aggregation can seldom be 100 % in our case owing to the equilibrium constants involved in imine formation. Normalization between 0 and 1 thus represents a way to demonstrate relative growth.

Fitting of photo-physical measurement data:

The kinetic traces obtained from absorption measurements were fit to Finke-Watzky's two state model for unseeded growth and Zhao and Moore's equation[6] for seeded growth.[7] Nucleation rate constants and rate of active termini were thus extracted from respective equations.

We use Finke-Watzky's model to simplify our analysis by deconvoluting the whole process in two rate constants. Rate of nucleation k_n and Rate of elongation k_e. Rate

of nucleation k_n includes imine formation and supramolecular nucleation, and as we show by experiments containing nucleation (Fig 2 c-e) and growth rate dependence with varying alkyl chains (Fig 2 d, that these two rates (imine formation and supramolecular nucleation) are co-dependent and inseparable in our case.

Viscosity measurements:

Viscosity measurements were performed on Anton Paar MCR 302 rheometer (air bearing). The measuring system was CP50-1 and the measuring cell configuration was P-PTD 200/80/Air. The gap between the surface and cone plate was 0.101 mm. Viscosity was measured over time at a constant shear rate of 50 Hz. Temperature was kept constant at 25 ^{o}C. A dead time (Time between sample loading and starting of measurement by the machine) of around 180 seconds is present in all measurements

pH measurements:

pH was measured by Malvern control dynamics pH meter. pH values were recorded manually at defined time periods and then plotted into trends.

NMR measurements:

NMR spectra were obtained with a Bruker AVANCE 400 (400 MHz w.r.t. ^{1}H nuclei) Fourier transform NMR spectrometer with chemical shifts reported in parts per million (ppm).

Dynamic light scattering measurements:

The measurements were carried out using a NanoZS (Malvern UK) employing a 532 nm laser at a back scattering angle of 173°. The samples were measured in a 10 mm glass cuvette. A dead time (Time between sample loading and starting of measurement by the machine) of around 45 seconds is present in all measurements.

Field Emission Scanning Electron Microscopy (FE-SEM) measurements:

FE-SEM measurements were performed on NOVA NANO SEM 600 (FEI) operated at 15 kV, by drop casting the solution on glass substrate. All samples were recorded in low vacuum mode (FE-SEM).

Transmission Electron Microscopy (TEM) measurements:

TEM measurements were performed on a JEOL, JEM 3010 operated at 300 kV. Samples were prepared by placing a drop of the solution on carbon coated copper grids followed by drying at room temperature. The images were recorded with an operating voltage of 300 kV.

Cryo-TEM measurements:

To study the length of fibres at different time points, samples were prepared using FEI Vitrobot system by plunge freezing in liquid ethane to preserve the native structure. Following are the parameters that were used: Blot time (s) – 1.0, Blot force – 0, Wait time (s) – 1.0, Blot total – 1, Drain time (s) – 0.5. The temperature was

maintained at 22 °C and humidity was > 90 %. Holey carbon grids were used for sample preparation bought from EMS. Imaging was done using Tecnai G2 Spirit Bio-TWIN Transmission Electron Microscope at 100 kV.

Analysis of cryo-TEM data:

Length analysis of the cryo-TEM images was done using a freeware imageJ. A frequency statistics was done on the obtained lengths and their number (L_n) and weighted average (L_w) was calculated. PDI was calculated as L_w/L_n. Following equations were used for number (L_n) and weighted average (L_w) calculation.

$$L_n = \frac{\sum_{i=1}^{n} N_i L_i}{\sum_{i=1}^{n} N_i} \qquad L_w = \frac{\sum_{i=1}^{n} N_i L_i^2}{\sum_{i=1}^{n} N_i L_i}$$

N_i is the frequency of length L_i.

Supplementary figures:

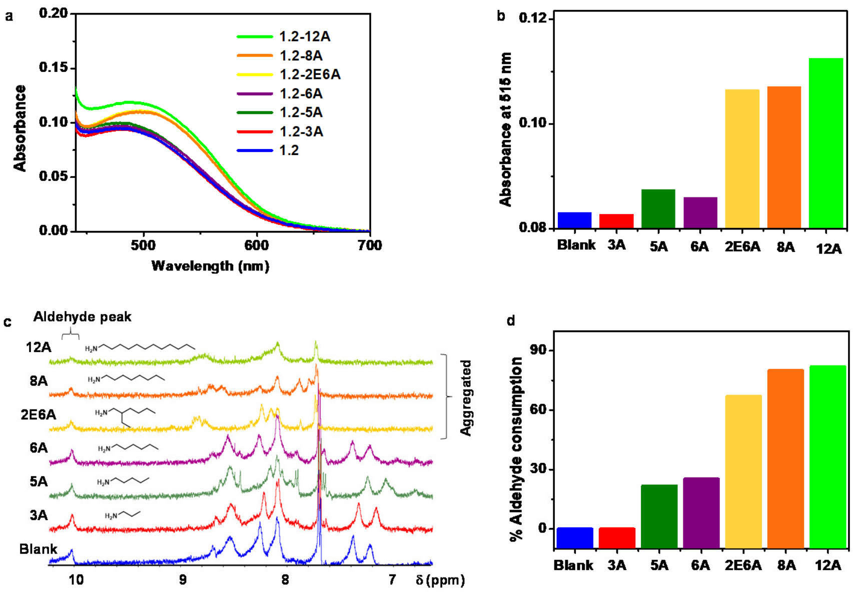

Supplementary Figure 3. a. Absorption spectra of **1.2** with various amines. **b.** Graph showing the extent of assembly with respect to each amine. ([**1**] = [**2**] = 1 mM, [alkyl amine] = 1 eq., pH = 11.0). **c.** ^{1}H-NMR of **1.2** with various amines, **d.** Graph showing the extent of aldehyde consumption with respect to each amine. ([**1**] = [**2**] = 1 mM, [alkyl amine] = 1 eq., pH = 11.0, D_2O).

Supplementary Note 1: In a typical ^{1}H-NMR experiment the required amount of **1** and **2** were mixed and 1.0 eq. of alkyl amine was added to it. The resulting solution was incubated for 1 hour at 25 °C. Blank solution in which no amine was added was also incubated under same conditions and duration before measurement. 1 mM of sodium acetate was added in each sample as an internal standard as acetate peaks come in an exclusive region (2.5

ppm). Considering the NMR spectra of each of these samples the consumption of aldehyde was measured against the internal standard. A brief look at even the blank sample shows merged, broad peaks and thus aldehyde consumption became necessary to be recorded by an internal standard as imine protons were indistinguishable from the merged peaks.

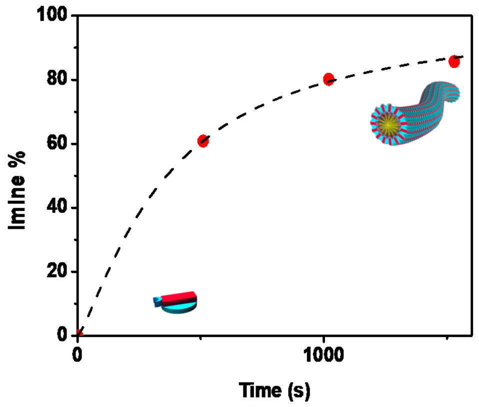

Supplementary Figure 4. ^{1}H-NMR monitored for kinetics of formation of **1.2-8A**.

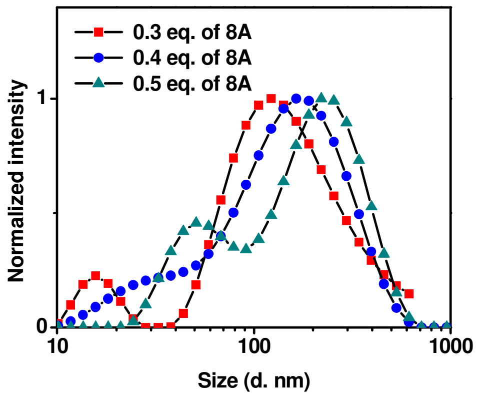

Supplementary Figure 5. DLS trend of **1.2** with various equivalents of **8A**, ([**1**] = [**2**] = 1 mM, pH = 11.0 buffer).

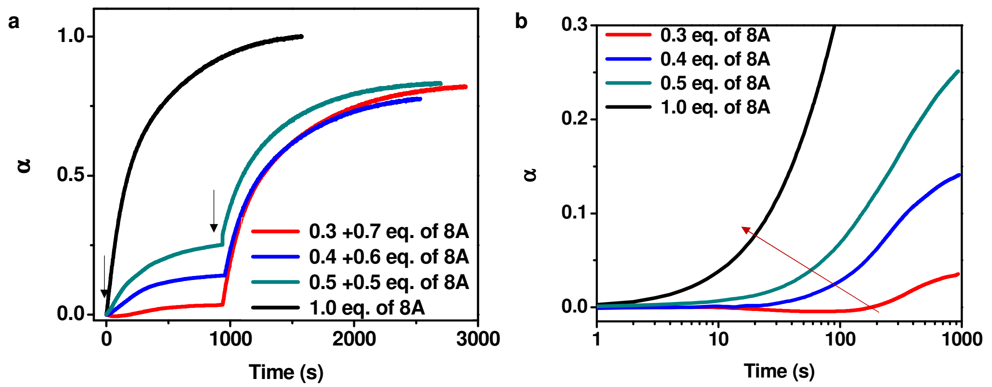

Supplementary Figure 6. a. Absorption trend of **1.2** with 1.0 eq of **8A** in batches followed at 515 nm, black arrow signifies addition of **8A** ([**1**] = [**2**] = 1 mM, pH = 11.0 buffer). **b**. Unseeded first half in figure **4a** on log scale, elucidating the different lag phases.

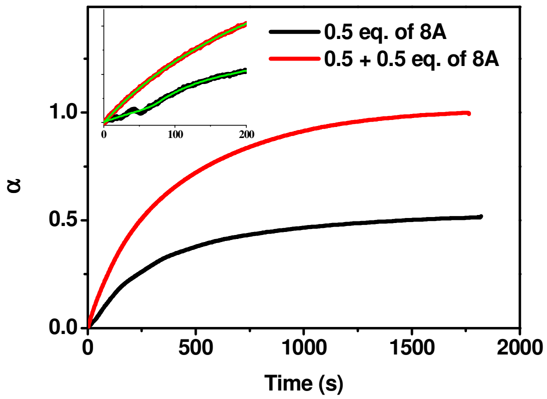

Supplementary Figure 7. Overlay of absorption trend of **1.2-8A** growth with 0.5 eq. of **8A** added in subsequent batches ([**1**] = [**2**] = 1.0 mM, pH = 11.0). Inset is a close up of earlier time points with green line as the smoothened overlay.

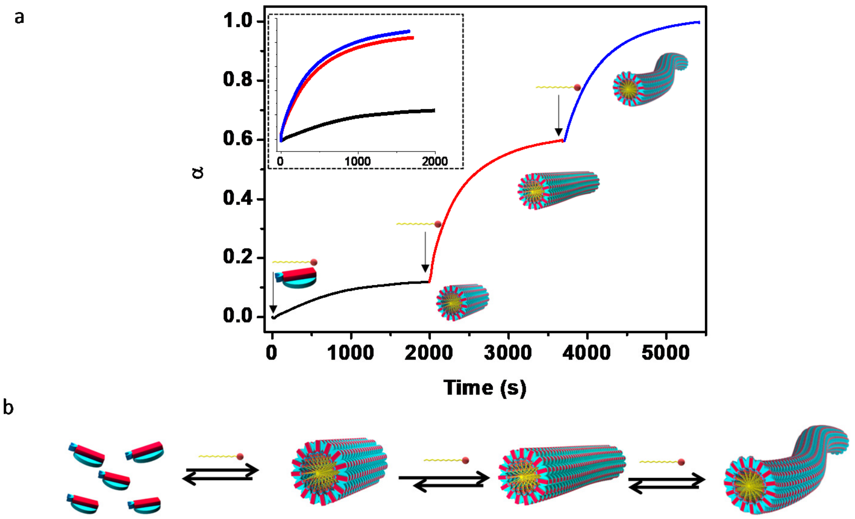

Supplementary Figure 8. a. Absorption trend of **1.2** with 0.99 eq. of **8A** in 3 batches followed at 515 nm, black arrow signifies addition of a new batch ([**1**] = [**2**] = 1 mM, pH = 11.0 buffer). Inset shows the overlay of the trend of 0.33 eq. (green) + 0.33 eq. (red) + 0.33 eq. (blue) batch showing difference between seeded and un-seeded growth. **b.** Schematic representation of the sequential growth experiment.

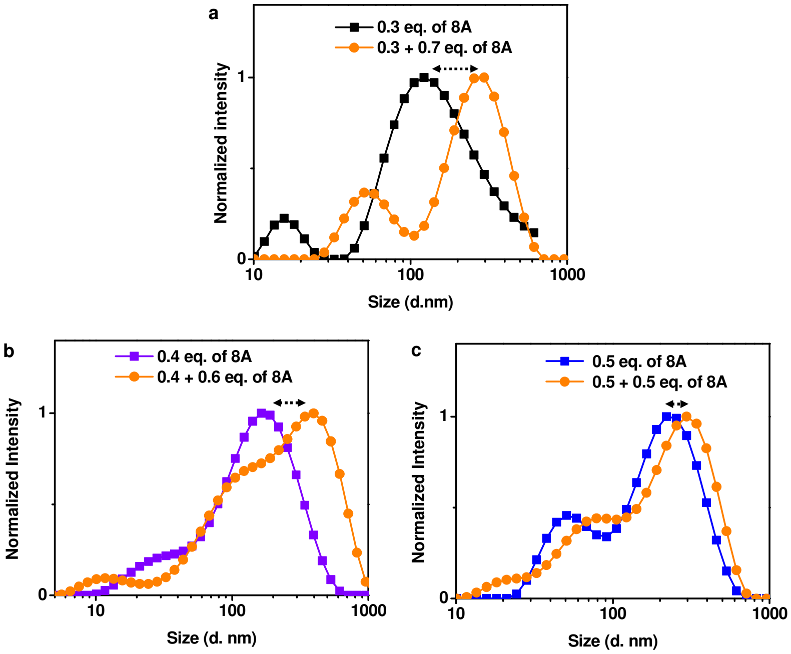

Supplementary Figure 9. a-c. DLS trend of **1.2** with 1.0 eq of **8A** in batches followed by DLS, black double arrow points at the decreasing difference between maximum size ([**1**] = [**2**] = 1 mM, pH = 11.0 buffer).

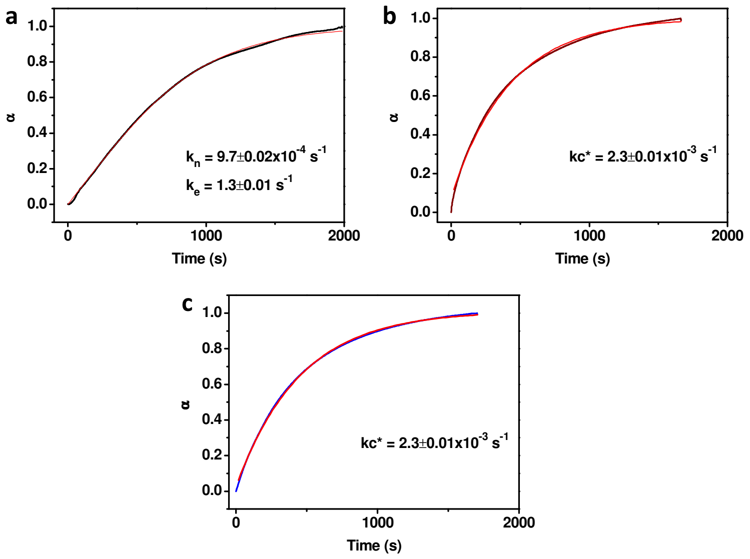

Supplementary Figure 10. Kinetic fits for the **a.** unseeded and **b.**, **c.** seeded growth traces on sequential addition of 0.3 eq. **8A** in **1.2**. (R^2> 0.99 in all fits)

Supplementary Note 2: a. is fitted to nucleation-elongation Finke-Watzky equation for nucleation (k_n) and elongation (k_e) rates. **b** and **c.** is fitted to Zhao and Moore's equation to extract rate of seeding by "active termini" of fibers. Since the rates are same, we believe number of active termini remain constant and hence a seeded polymerization with negligible independent nucleation occurs.

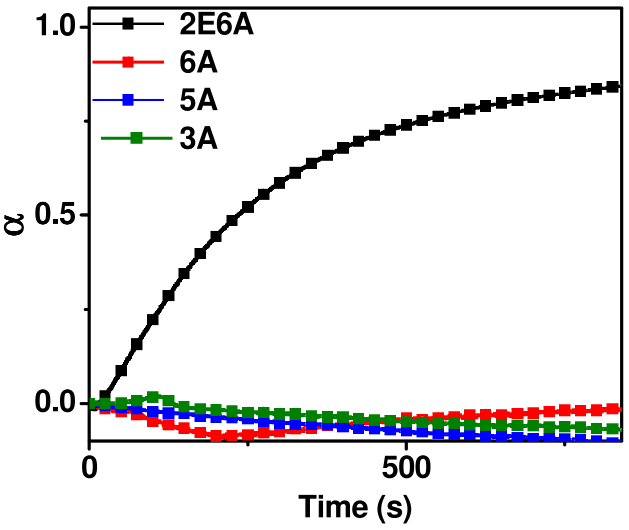

Supplementary Figure 11. Absorption trend of **1.2** with various amines followed at 515 nm, ([**1**] = [**2**] = 1 mM, [various amine] = 0.5 eq., pH = 11.0 buffer)

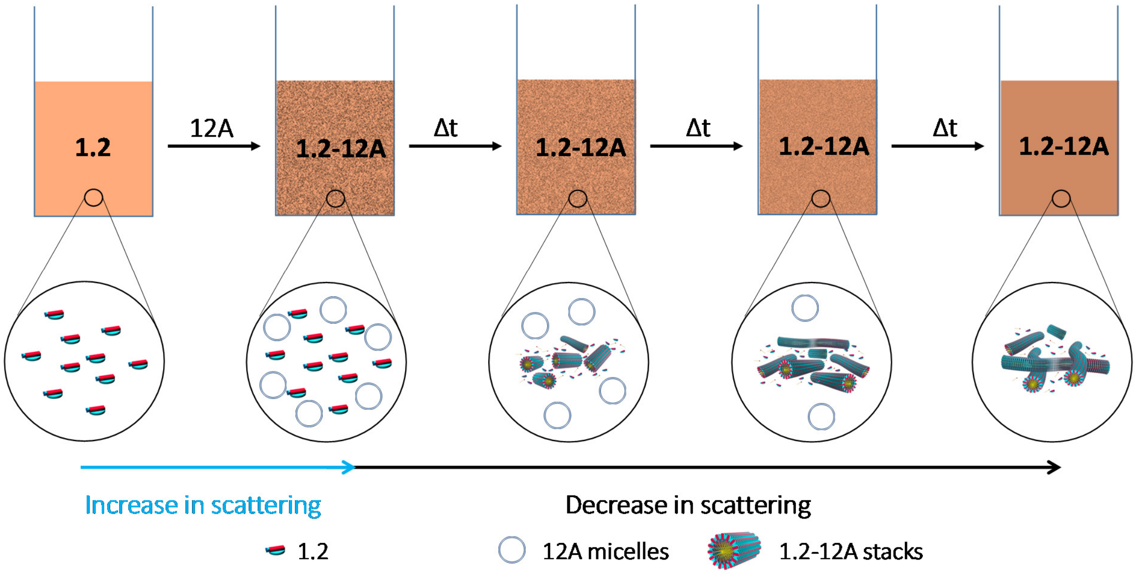

Supplementary Figure 12. Schematic showing the evolution of scattering over time in **1.2-12A** formation.

Supplementary Note 3: Most of the studies in this article were done on **8A** or its lower homologue. These amines do not have a considerable formation of independent micelles of their own and therefore are a good homogenous source in aqueous solution. However one must consider the question that what would be the scenario if alkyl amines formed their own micelles as well.

To study such a scenario we analysed the same **1.2** with *n*-dodecyl amine (**12A**) instead of *n*-octyl amine (**8A**). We observed that as soon as **12A** is added to the solution containing **1.2** an opaque suspension is formed (due to the micelle formation in water). This suspension clears over time resulting in imine bound aggregate.

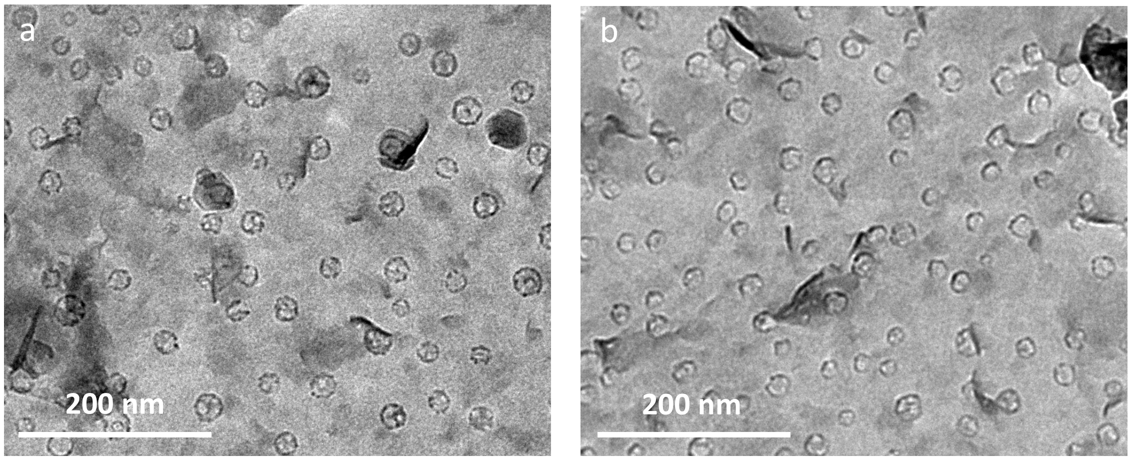

Supplementary Figure 13. a-b. TEM images of **12A** micelles in water. ([**12A**] = 1 mM, pH = 11).

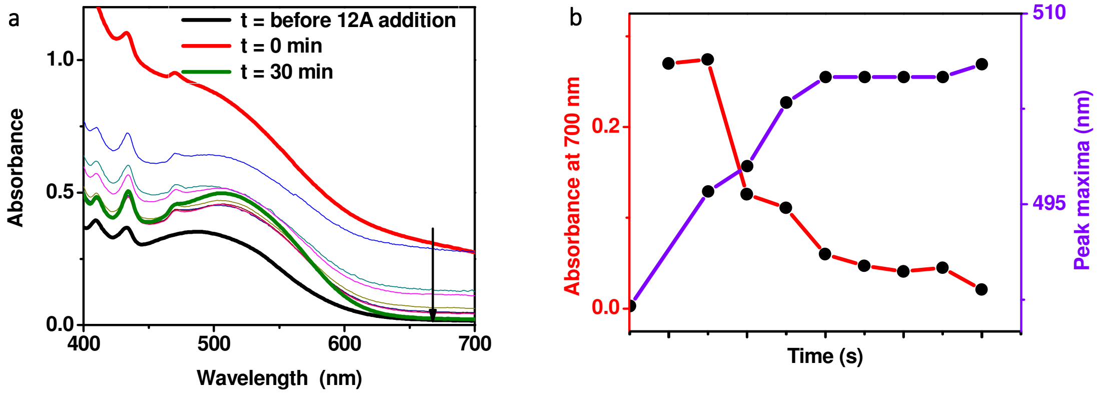

Supplementary Figure 14. a. Absorption spectra of **1.2** with 1.0 eq of **12A**, black arrow signifies decrease in scattering ([**1**] = [**2**] = 1 mM, pH = 11.0 buffer). **b.** Overlay of the trends of change in wavelength maxima and scattering over time from spectra in **a**.

Supplementary Note 4: We followed the growth process of **1.2-12A** with absorption spectra which confirmed the instantaneous increase of scattering on **12A** addition and then subsequent gradual decrease. It should be noted that the final spectra has very less scattering and has absorption features resembling that of an imine based aggregate (increased intensity of CT band) .We plotted the shift in maxima wavelength with time (signifies imine based aggregation) and compared it to the scattering changes. To our surprise both these kinetics are very similar with shift in peak maxima wavelength signifying imine based aggregation and hence increases with time. On the other hand decrease in scattering suggests decrease in population of independent micelles of **12A** and hence represent consumption of **12A** into an imine based aggregate. Both kinetics hence represent the same process.

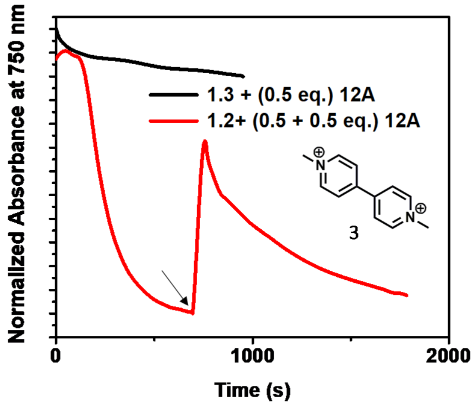

Supplementary Figure 15. Absorption trend of **1.2** and **1.3** with 0.5 eq of **12A**, black arrow signifies addition of another batch of **12A** ([**1**] = [**2**] = [**3**] = 1 mM, pH = 11.0 buffer). Inset shows the molecular structure of **3**.

Supplementary Note 5: To confirm our hypothesis we performed the same study with Viologen moiety devoid of an aldehyde group (**3**). We followed the scattering trace of **12A** at

750 nm. On addition of **12A** in a solution containing **1.3** no decrease in scattering was observed. However in case of **1.2** a decrease was observed but was accompanied with initial lag phase. To this subsequent solution further aliquot of **12A** was added and immediate decay of scattering was observed without a lag phase. This study is representative of various important points. First being the decrease in scattering is indeed due to **12A** consumption to form **1.2-12A**. The fact that first aliquot addition has a lag phase however the second aliquot does not; is reminiscent of the seeded growth observed in case of **8A**. We hypothesize that when **12A** is added into the solution it forms its own micelles (Supplementary Fig. 11). However overtime **12A** begins to get consumed into **1.2-12A** aggregates. The lag phase is perhaps a representation of another kinetic barrier that gets introduced due to mass transfer from **12A** micelles to **1.2-12A** via dissolution. However when the second aliquot is added the activated nuclei that already exist consume **12A** at a much faster rate hence the disappearance of the lag phase. Additional lag phase makes sure that rate of formation of **1.2-12A** nuclei is slow. A slower growth due to additional kinetic barrier thus forms for a good system to study living supramolecular polymerization as changes in kinetics are more evident. Further query arises in regards to what method should one follow to measure the growth trend of **1.2-12A**. Direct increase in CT band is not an option in this case due to the significant amount of scattering produced by **12A** micelles. Since as shown in previous discussion the decrease in **12A** scattering is representative of **1.2-12A** aggregation the growth has been monitored indirectly by **12A** consumption for further experiments. We performed sequential study of growth with various equivalents of **12A**.

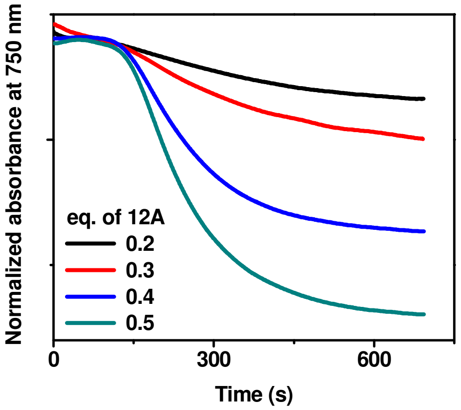

Supplementary Figure 16. a. Absorption trend of **1.2** with various equivalents of **12A**, ([**1**] = [**2**] = 1 mM, pH = 11.0 buffer). **b.** Manually displaced lag phases with arrows pointing towards the end of a lag phase at a particular equivalent of **12A**.

Supplementary Note 6: Similar to studies with **8A** this also showed increased rate and extent of aggregation with increase in the amount of **12A**. Also a closer look at the lag phase suggested the lag phase increased with increase in **12A** concentration, which is again suggestive of the fact that lag phase in this case is due to additional barrier of **12A** caused by its micelles.

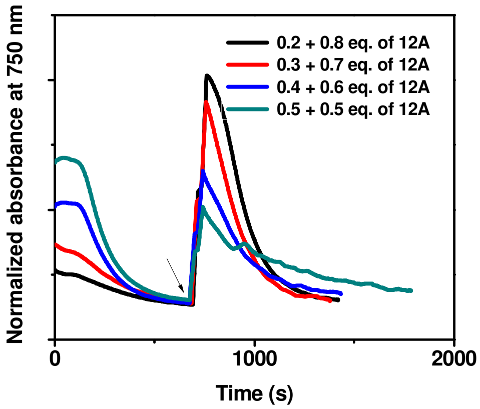

Supplementary Figure 17. Absorption trend of **1.2** with 1.0 eq of **12A** in various batches, black arrow signifies addition of another batch of **12A** ([**1**] = [**2**] = 1 mM, pH = 11.0 buffer).

Supplementary Note 7: We further went ahead to carry on seeding experiments on this system and similar to the case in **8A** we added the amines in batches. First batch clearly showed the presence of a lag phase which was absent in the second addition. Moreover one can notice that at higher percentages of seed (high first aliquot proportion) absolutely no trace of sigmoidal growth was noticed however slight sigmoidal nature appears at lower seed ratios (0.2). This signifies perhaps the limit of nuclei concentration that can give rise to a catalytic growth.

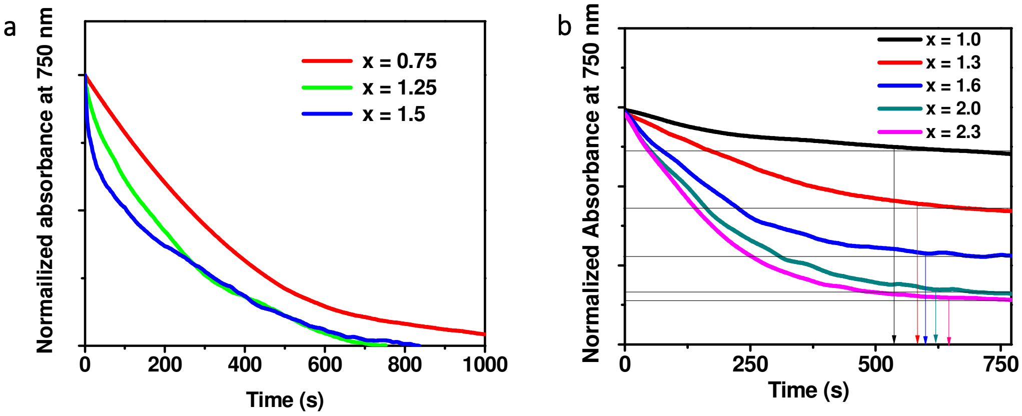

Supplementary Figure 18. a-b. Absorption trend of **1.2-12A** with x[N]+[M] and [N]+x[M] experiments respectively followed at 750 nm, ([**1**] = [**2**] = 1 mM, pH = 11.0 buffer). [N] = nuclei, [M] = incoming monomer, x = variable)

Supplementary Note 8: We also went ahead and performed nuclei and monomer variation experiments with **12A**. Increasing the percentage of nuclei clearly increased the rate of scattering decay which relates to rate of growth. Moreover when nuclei concentration was kept constant and monomer concentration was varied, higher monomer percentages took longer time to saturate. Both of these experiments reiterate the previous hypothesis of seeded self-assembly.

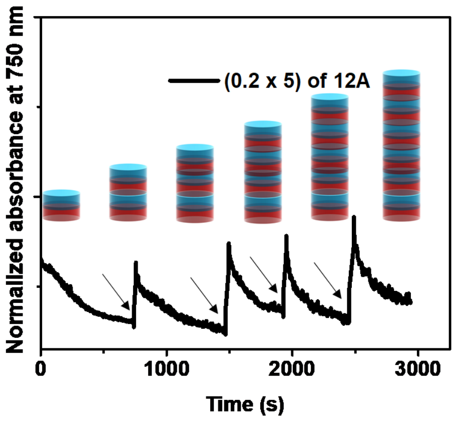

Supplementary Figure 19. Absorption trend of **1.2** with 1.0 eq of **12A** in 5 batches followed at 750 nm, black arrow signifies addition of a new batch ([**1**] = [**2**] = 1 mM, pH = 11.0 buffer). Schematic represents the sequential growth of stacks.

Supplementary Note 9: Furthermore we also demonstrated the sequential addition which in this case could be done up to 5 cycles (0.2x5). First cycle has a lag phase with a sigmoidal nature and the subsequent 4 cycles do not. This clearly suggests that these additions are indeed sequential.

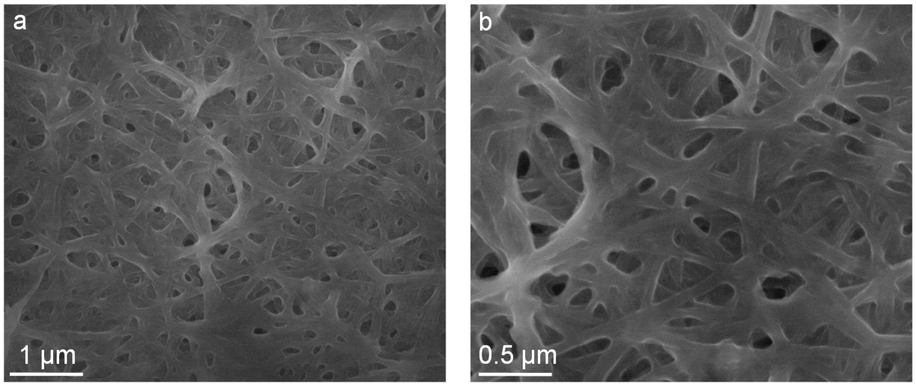

Supplementary Figure 20. a-b. FE-SEM images of **1.2** with 1.0 eq of **12A** ([**1**] = [**2**] = 1 mM, pH = 11.0 buffer).

Supplementary Note 10: Another important analysis was the morphology of the aggregate. FE-SEM analysis of **1.2-12A** clearly suggests linear aggregates. This is in accordance with the previously proposed growth model.

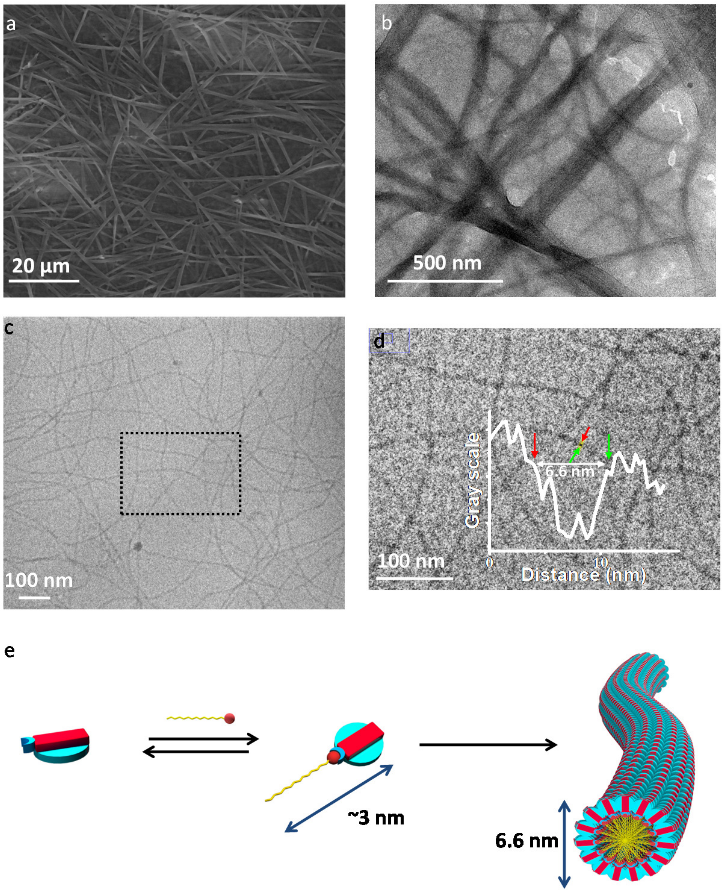

Supplementary Figure 21. a-c. FE-SEM, TEM and cryo-TEM respectively of **1.2** with 1.0 eq. of **8A**, ([**1**] = [**2**] = 1 mM, pH = 11.0 buffer). **d.** Zoomed region shown of black box marked in image **c.** Inset (**c**) Gray scale analysis to probe the width of the fiber. **e.** Schematic representation of the cylindrical micelle packing with molecular dimensions.

a

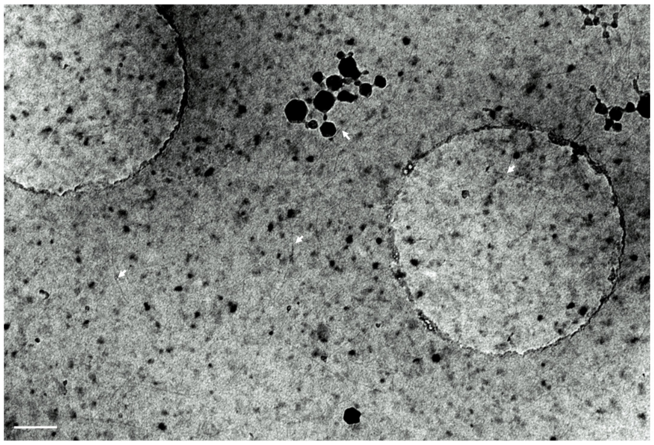

b

[M]/[S]	L_n	PDI (L_w/L_n)
0	186 nm	1.30
0.5	282 nm	1.20
1.0	435 nm	1.12
1.5	548 nm	1.10

Supplementary Figure 22. a. Cryo-TEM images of **1.2-8A** with seed (1 mM of **8A**) + monomer (1.5 mM of **8A**) ([**1.2**] = 2.5 mM). Scale bar: 200 nm. White arrows point to some of the fibres in the image. **b.** Table summarizing the average lengths and polydispersity indices obtained from various monomer to nuclei ratio.

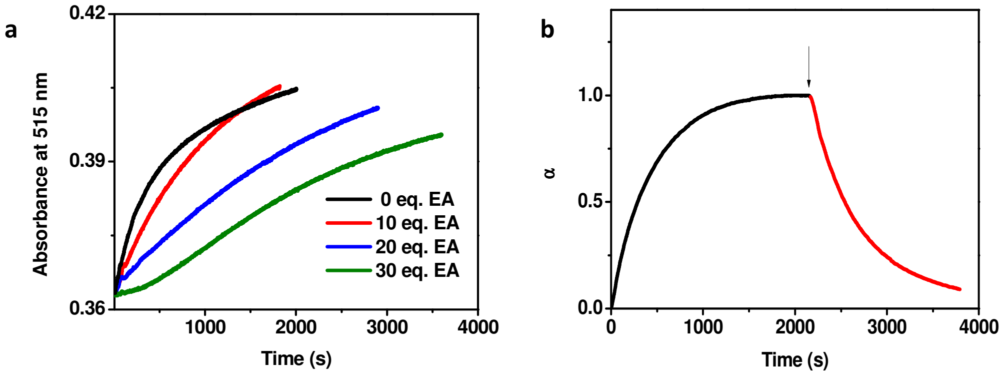

Supplementary Figure 23. a. Native UV-vis trend of **1.2-8A** growth with various equivalents of **EA**. ([**1**] = [**2**] = [**8A**] = 1 mM, 50 mM pH = 11 buffer) and **b.** UV-vis trend of **1.2** with 1.0 eq of **8A** (black trace) and subsequent decay on addition of 60.0 eq of **EA** (red trace), black arrow signifies addition of **EA** ([**1**] = [**2**] = 1 mM, 50 mM pH = 11 buffer).

In the Figure 3c, Supplementary Figure 21 the red curve (10 eq. EA) seems to take over the black curve (0 eq. EA). We hypothesize this is due to a co-assembly of some percentage between 1.2-EA and 1.2-8A, similar to experiments in Figure 2f. The delay in nucleation is however clear from the trends and respective kinetic fits (Supplementary Figure 22).

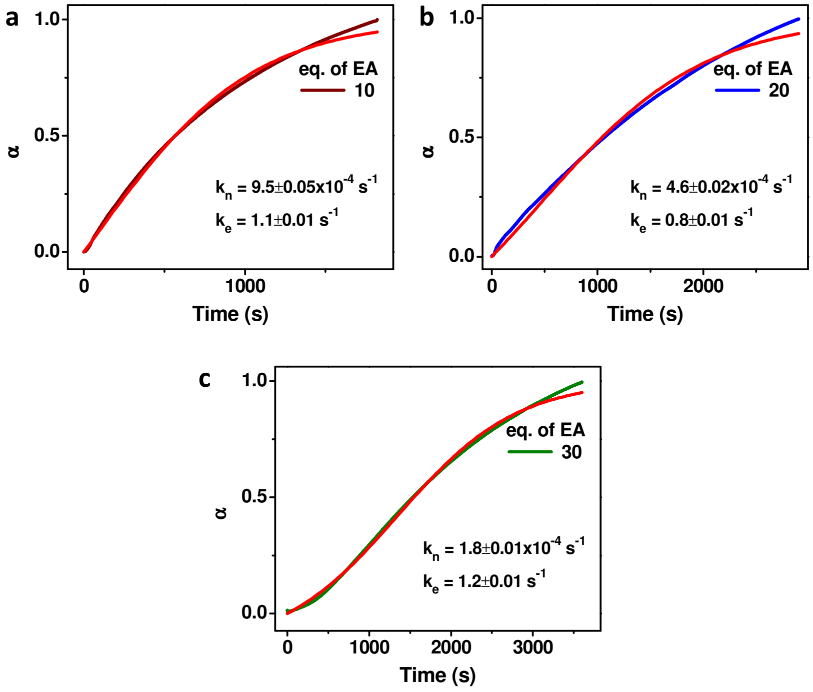

Supplementary Figure 24. Kinetic fits in Finke-Watzky equation for the nucleation rates at varying equivalents of EA in the unseeded growth of **1.2-8A**. (R^2> 0.99 in all fits)

Supplementary Note 11: A gradual decrease in nucleation (k_n) rates and insignificant change in and elongation (k_e) rate is observed elucidating the delayed nucleation by addition of EA.

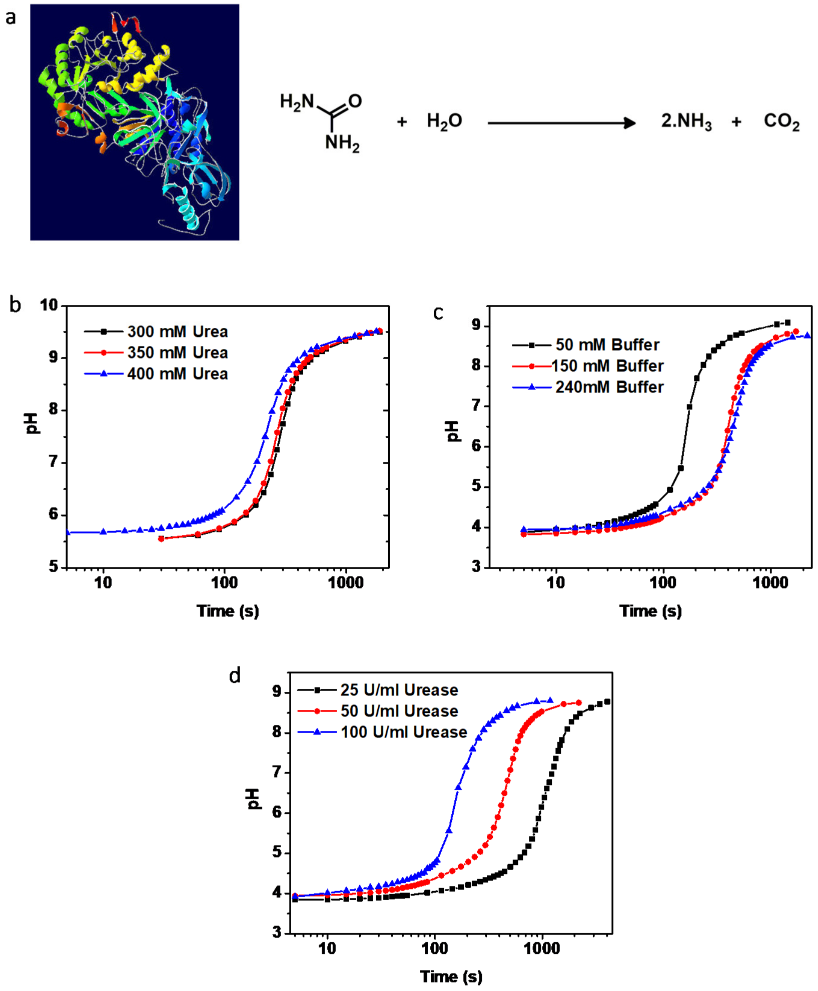

Supplementary Figure 25. a. Crystal structure of **Urease** accompanied by the chemical reaction representing the degradation of Urea by the same, **b-d.** Kinetic pH traces (b: [Urease] = 50 U, 240 mM pH = 4 buffer, c: [Urease] = 50 U, [Urea] = 400 mM, pH = 4 buffer, d: 240 mM pH = 4 buffer, [Urea] = 400 mM). (**a.** is copyrighted for free use, and has been taken from the following link https://commons.wikimedia.org/w/index.php?curid=104129).

Supplementary Note 12: Urease is an enzyme that acts on Urea in presence of water to release two molecules of ammonia and one molecule of carbon dioxide. As ammonia is formed it increases the pH of the solution gradually. Apart from changing the pH the activity of Urease itself is pH dependent with less reactivity at pH = 4, peaking at pH = 7, and finally decaying again with further increase in pH. Therefore from acidic to neutral this change is autocatalytic as it increases the enzyme kinetics and beyond neutral it is inhibitory as the activity of the enzyme then decreases. This phenomena makes pH changes an integral part of Urease kinetics.

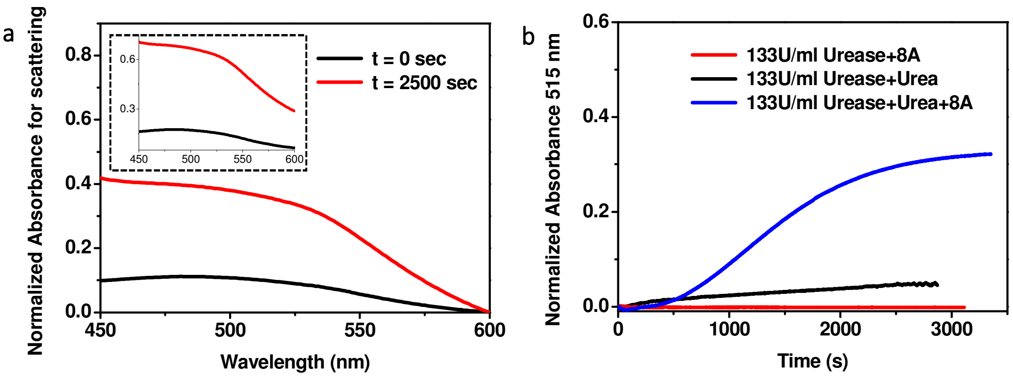

Supplementary Figure 26. a. Absorption spectra of **1.2-8A** evolution ([Urease] = 133 U, [Urea] = 400 mM, 200 mM pH = 7 buffer, [**1.2**] = 1mM, [**8A**] = 5 mM); inset shows the un-normalized Absorption spectra**, b.** Kinetic traces followed at 515 nm ([Urea] = 400mM, [**1.2**] = 1 mM, [**8A**] = 5 mM, 200 mM pH = 7 buffer).

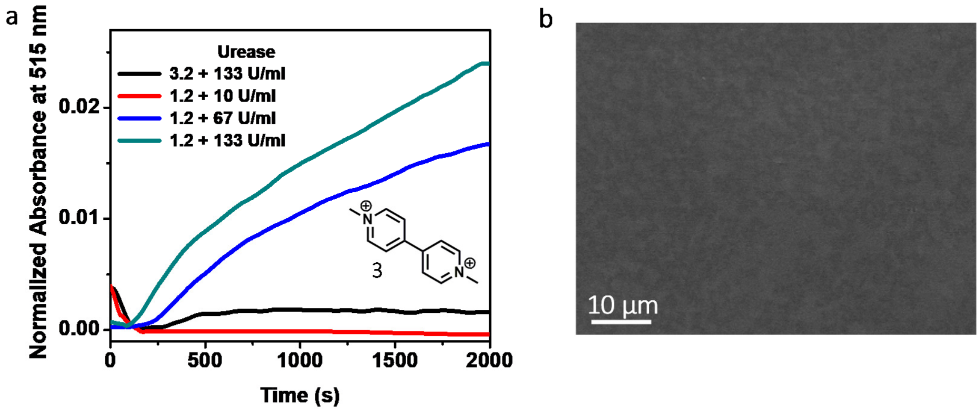

Supplementary Figure 27. a. Kinetic traces followed at 515 nm. Black trace represents Urease addition to a solution containing **1.3** and **8A**. Other traces (red, green and blue) represent Urease addition to **1.2**. Structure of **3** is shown in the inset. ([Urea] = 400 mM, [**1.2**] = 1 mM, [**8A**] = 5 mM, 200 mM pH = 7 buffer) and **b.** FE-SEM image at the saturation point of the sample containing [Urea] = 400 mM, [**1.2**] = 1 mM, [Urease] = 133 U. This is general representation of the sample signifying no self-assembled structures were observed.

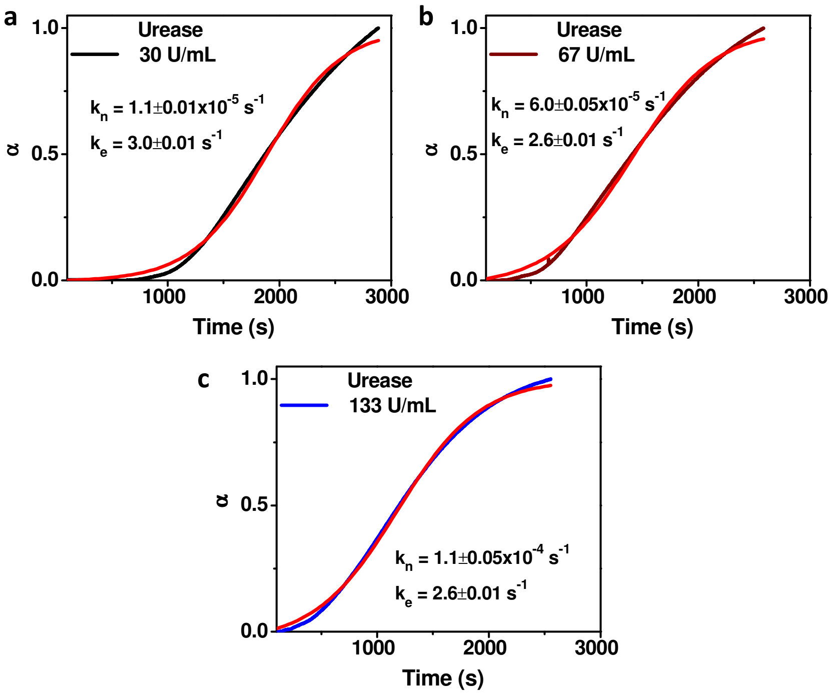

Supplementary Figure 28. Kinetic fits in Finke-Watzky equation for the nucleation rates at varying units of urease in the unseeded growth of **1.2-8A**. (R^2> 0.99 in all fits)

Supplementary Note 13: A gradual decrease in nucleation (k_n) rates and insignificant change in and elongation (k_e) rate is observed elucidating the delayed nucleation by addition of various units of urease.

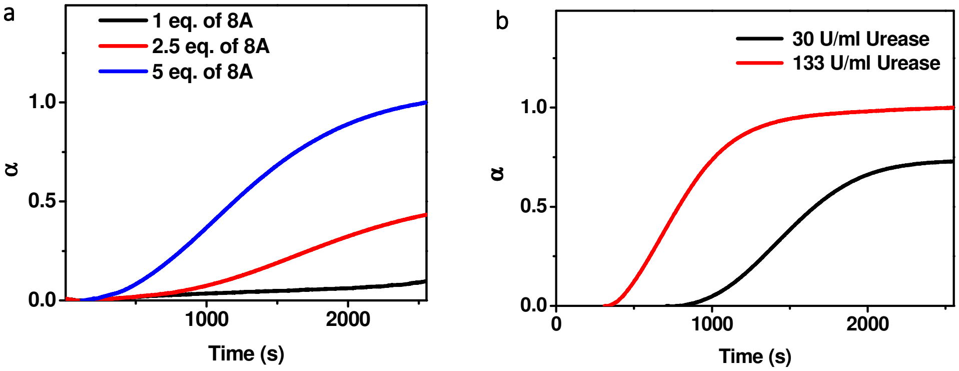

Supplementary Figure 29. a. Kinetic traces of **1.2-8A** growth followed at 515 nm with variation of [**8A**] **b.** Kinetic traces of **1.2-8A** growth followed at 650 nm with variation of [Urease] (a: [Urea] = 400 mM, [**1.2**] = 1 mM, [Urease] = 133 U, 200 mM pH = 7 buffer, b: [Urea] = 400 mM, [**1.2**] = 1 mM, [**8A**] = 5 mM, 200 mM pH = 7 buffer).

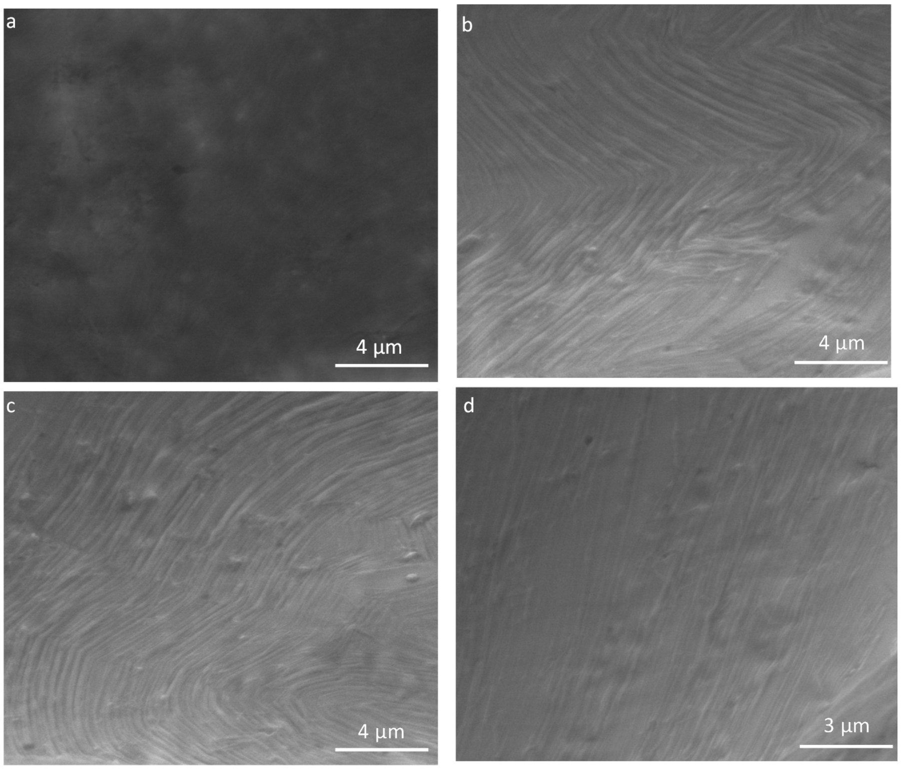

Supplementary Figure 30. a-d. FE-SEM images showing elongated linear fibers ([Urea] = 400 mM, [**1.2**] = 1 mM, [Urease] = 133 U, [8A] = 5 mM, 200 mM pH = 7 buffer). The structures were highly condensed and bundled. Prospective reasons could by the high excess of ammonia and urea in solution. The final pH (~9) could also be a prospective factor as this is close to pKa's of reaction components.

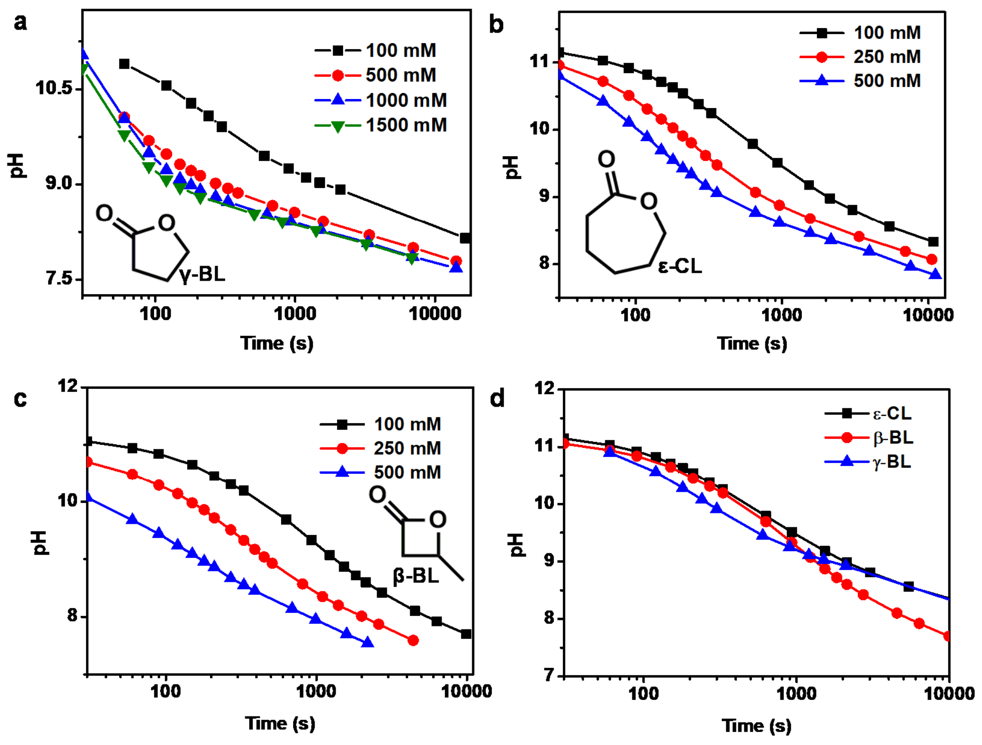

Supplementary Figure 31. a-c. Kinetic pH traces of various concentrations of different lactones (50 mM, pH = 11 buffer) and **d.** Comparative pH traces of various lactones [lactone] = 100 mM.

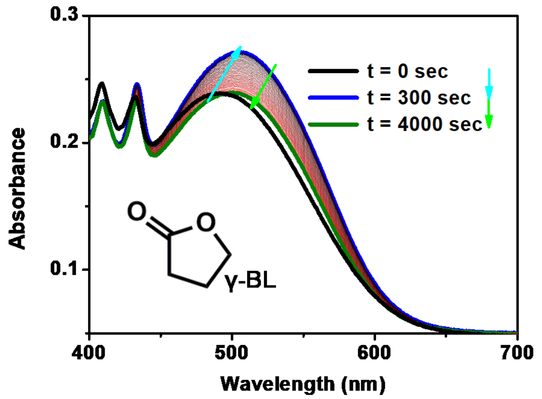

Supplementary Figure 32. Absorption spectra following the changes in **1.2-8A** absorption over time ([**γ-BL**] = 250 mM, pH = 11 buffer, [**1.2**] = 1 mM, [**8A**] = 5 mM).

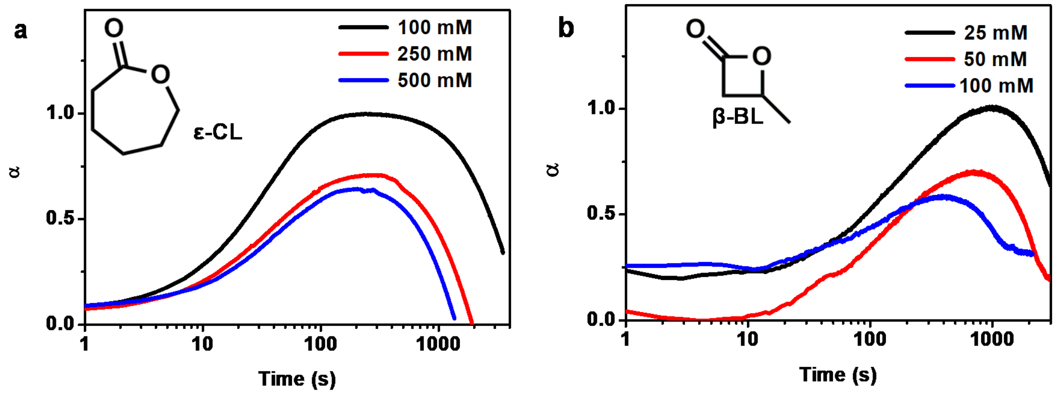

Supplementary Figure 33. a-b. Temporal profile of **1.2-8A** growth various concentrations ε-Caprolactone (**ε-CL**) and β-Butyrolactone (**β-BL**) respectively ([**1**] = [**2**]= 1 mM, [**8A**] = 5 mM, pH = 11).

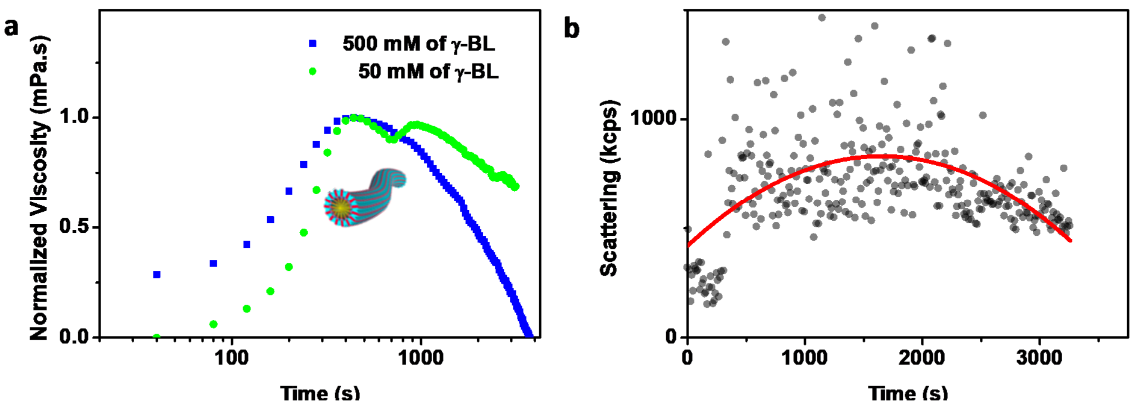

Supplementary Figure 34. a. Transiency followed via viscosity measurement at various concentrations of **γ-BL** ([**1**] = [**2**] = 1 mM, [**8A**] = 5 mM, pH = 11). **b.** Transient trace followed over time via DLS (red polynomial fit to follow the eye) ([**γ-BL**] = 500 mM, 50 mM, pH = 11 buffer, [**1.2**] = 1 mM, [**8A**] = 5 mM). A non-linear trend suggesting growth and decay is clearly visible.

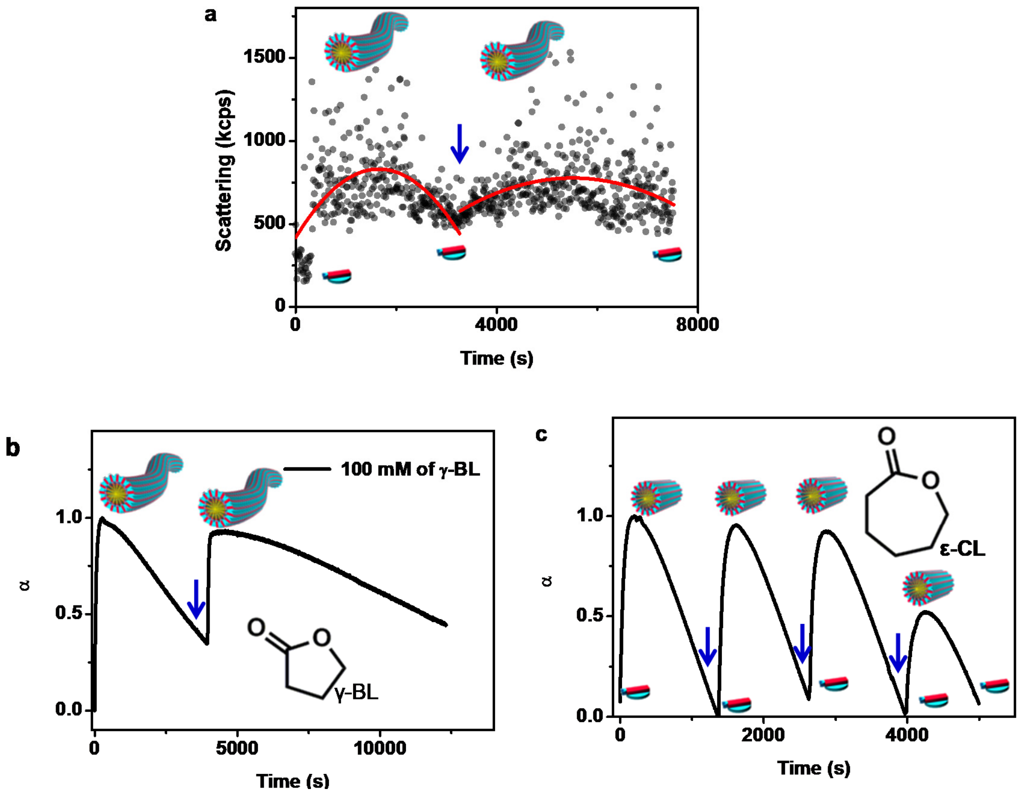

Supplementary Figure 35. a. Transient refueling traces followed over time through DLS (red polynomial fit to follow the eye, blue arrow indicates conc. NaOH addition) ([**γ-BL**] = 500 mM, 50 mM, pH = 11 buffer, [**1.2**] = 1 mM, [**8A**] = 5 mM). **b.** Transient refueling traces followed over time via Absorption (blue arrow indicates conc. NaOH addition) ([**γ-BL**] = 100 mM, [**1**] = [**2**]= 1 mM, [**8A**] = 5 mM, pH = 11). **c.** Transient refueling traces followed over time via Absorption (515 nm)(blue arrow indicates conc. NaOH addition) ([**ε-CL**] = 500 mM, 50 mM pH = 11 buffer, [**1.2**] = 1 mM, [**8A**] = 5 mM).

Supplementary Note 14: We used 5 µl of 1.6 M NaOH as the refuelling agent. For 500 mM of ε-CL indeed due to the faster kinetics we could go up to four cycles of transient assemblies. The fourth cycle however lesser amplitude has and also is slightly slower as compared to previous cycles. This again could be due to consumption of lactone and an added fact that same amount of NaOH cannot revive the same amount of amines as the shear consumption of hydroxyl ions pile up.

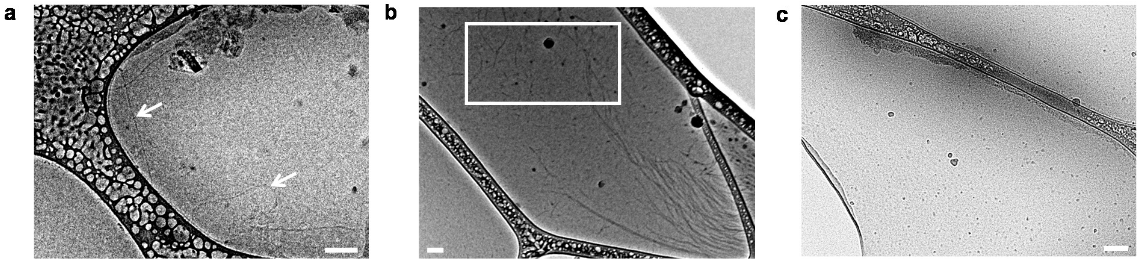

Supplementary Figure 36. a-c. Cryo-TEM images of **1.2-8A** with 500 mM ε-Caprolactone (**ε-CL**) at 300 secs, 1980 secs and 14400 secs respectively. White arrows in **a.** indicate linear aggregates, white box in **b** indicate broken aggregates. Scale for a: 100 nm, b: 100 nm, c: 200 nm.

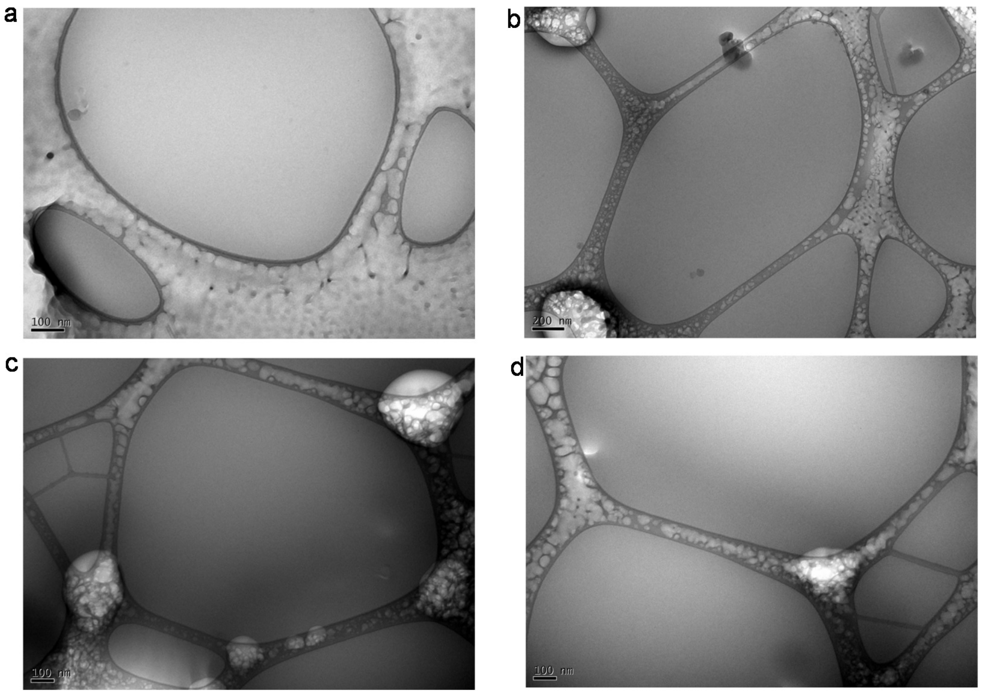

Supplementary Figure 37. a-d. Cryo-TEM images of **1.2-8A** with 500 mM ε-CL at 43200 secs. (pH = 11 buffer, [**1.2**] = 1 mM, [**8A**] = 5 mM).

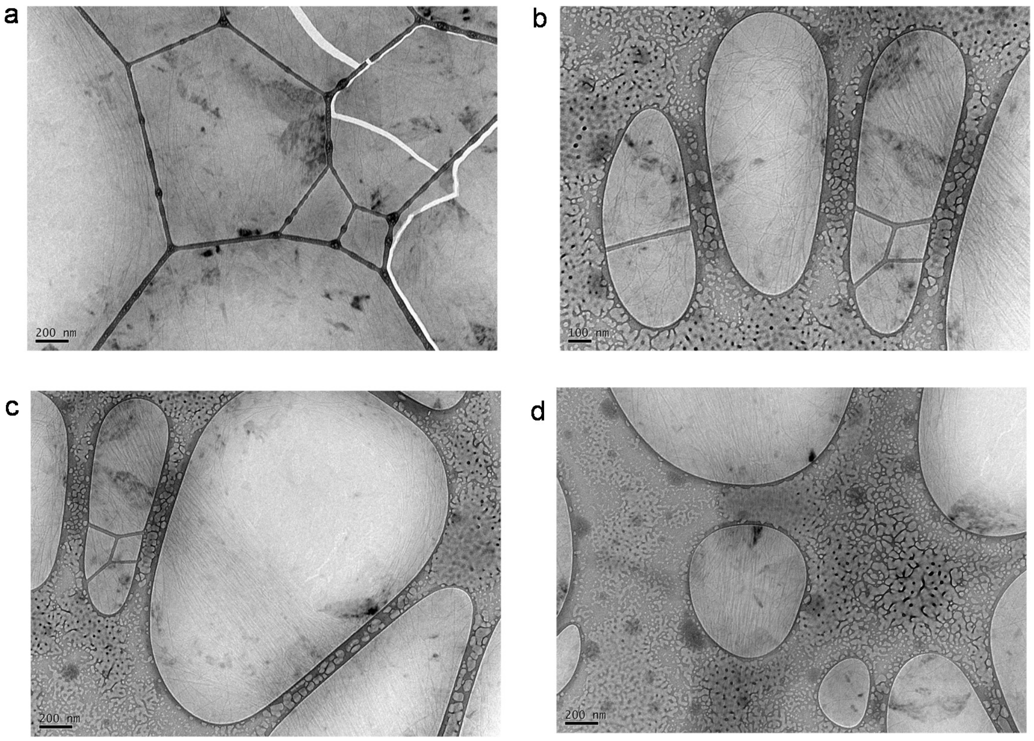

0

Supplementary Figure 38. a-d. Cryo-TEM images of **1.2-8A** without lactone at 12 hrs. (pH = 11 buffer, [**1.2**] = 1 mM, [**8A**] = 5 mM).

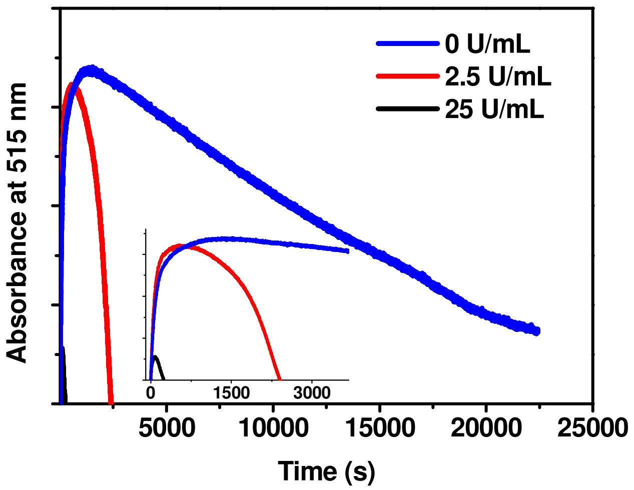

Supplementary Figure 39. Native temporal profile of **1.2-8A-est** growth at various concentrations of lipase ([**1**] = [**2**]= 1 mM, [**8A-est**] = 2 mM, pH = 11). (Inset shows changes at smaller time scales)

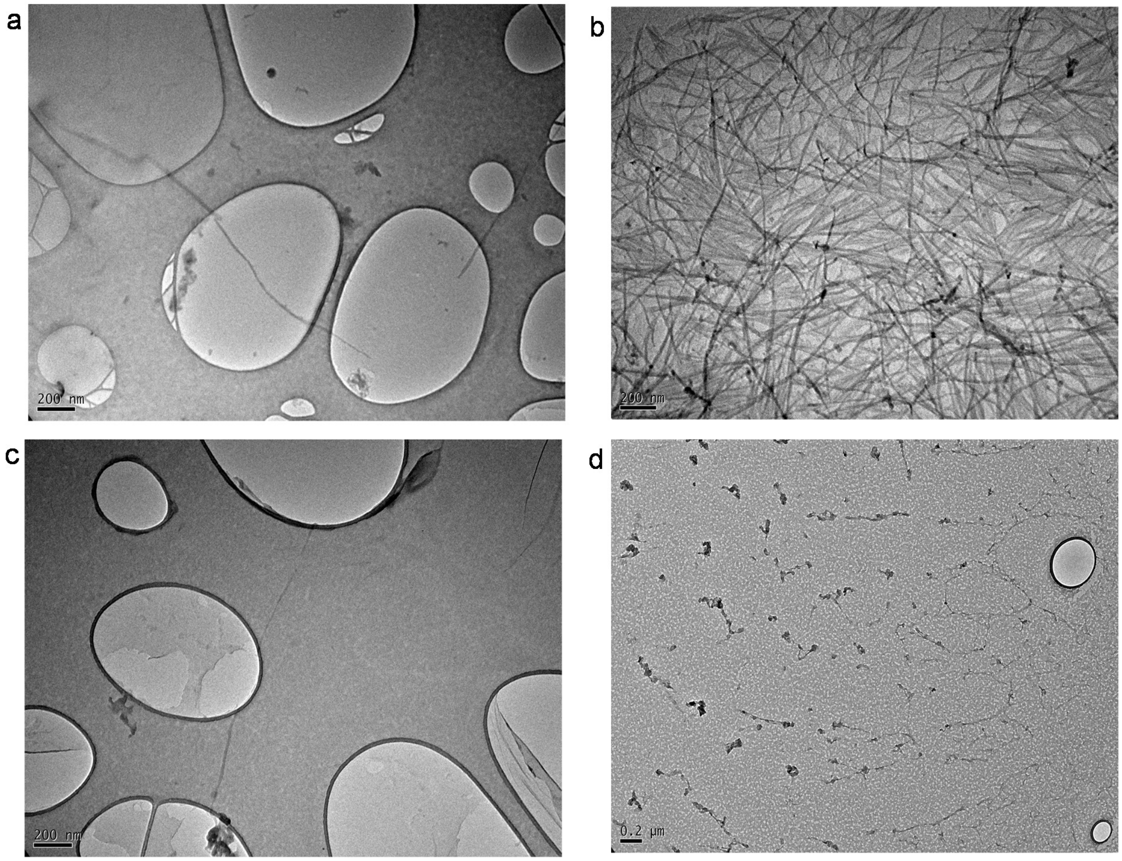

Supplementary Figure 40. a-d. Cryo-TEM images of **1.2-8A-est** at 1, 2, 4 and 24 hrs respectively. (pH = 11, [**1.2**] = 1 mM, [**8A-est**] = 2 mM, [**Lipase**] = 0 U/ml).

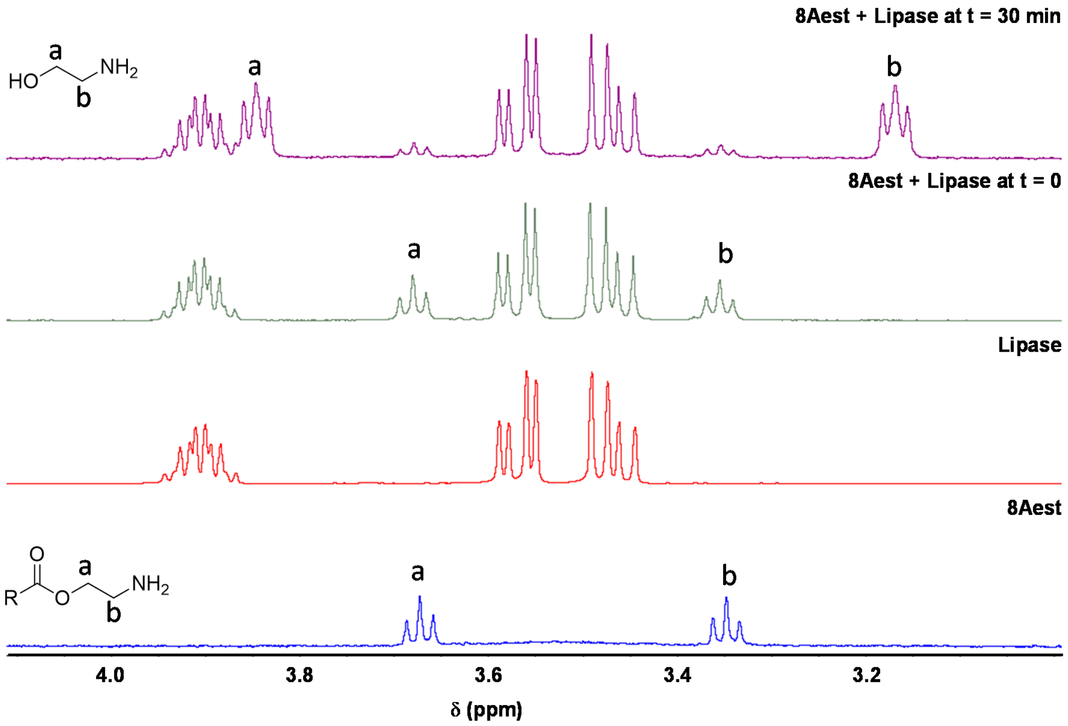

Supplementary Figure 41. ^{1}**H NMR** of **8A-est** and lipase at t = 0 and 30 min. (pH = 11, [**1.2**] = 1 mM, [**8A-est**] = 2 mM, [**Lipase**] = 2.5 U/ml). The peaks at δ(ppm) 3.67 and 3.34 corresponding to alkyl protons of ethanol amine are shifted to 3.84 and 3.16 confirming the hydrolysis of ester group by lipase at pH = 11.

Supplementary References:

1. http://delloyd.50megs.com/moreinfo/buffers2.html

2. Ghosh, A., Rao, K. V., George, S. J. & Rao, C. N. R. Noncovalent Functionalization, Exfoliation, and Solubilization of Graphene in Water by Employing a Fluorescent Coronene Carboxylate. *Chem. Eur. J.* **16**, 2700-2704 (2010).

3. Rao, K. V., Jayaramulu, K., Maji, T. K. & George, S. J. Supramolecular hydrogels and high-aspect-ratio nanofibers through charge-transfer-induced alternate co-assembly. *Angew. Chem. Int. Ed.* **49**, 4218-4222 (2010).

4. Yamaguchi, H. & Harada, A. Supramolecular Formation of Antibodies with Viologen Dimers: Utilization for Amplification of Methyl Viologen Detection Signals in Surface Plasmon Resonance Sensor. *Biomacromolecules* **3**, 1163-1169 (2002).

5. Chatterjee, S. & Ramakrishnan, S. A novel photodegradable hyperbranched polymeric photoresist. *Chem. Commun.* **49**, 11041-11043 (2013).

6. Zhao, D. & Moore, J. S. Nucleation–elongation: a mechanism for cooperative supramolecular polymerization. *Org. Bio. Chem.* **1**, 3471-3491 (2003).

7. Morris, A. M., Watzky, M. A. & Finke, R. G. Protein aggregation kinetics, mechanism, and curve-fitting: a review of the literature. *Biochim. Biophys. Acta, Proteins Proteomics* **1794**, 375-397 (2009).
